# BNIP3-mediated mitophagy aggravates placental injury in preeclampsia via NLRP1 inflammasome

**DOI:** 10.3389/fimmu.2025.1530015

**Published:** 2025-04-02

**Authors:** Man Zhao, Zexin Yang, Yan Kang, Zhenya Fang, Changqing Zhang, Chunying Wang, Meijuan Zhou, Junjun Guo, Anna Li, Meihua Zhang

**Affiliations:** Key Laboratory of Maternal & Fetal Medicine of National Health Commission of China, Shandong Provincial Maternal and Child Health Care Hospital Affiliated to Qingdao University, Jinan, China

**Keywords:** preeclampsia, trophoblast, mitophagy, BNIP3, NLRP1

## Abstract

**Introduction:**

Preeclampsia (PE) is a hypertensive disorder of pregnancy characterized by pronounced placental oxidative stress and inflammatory damage. However, the contribution of mitophagy to inflammation-induced placental injury in PE remains unclear.

**Methods:**

Human placenta samples were collected from 15 normal pregnant women and 15 preeclampsia pregnant women. Protein expression was analyzed by western blotting, while immunofluorescence staining was employed to localize inflammatory mediators. Mitochondrial reactive oxygen species were quantified using MitoSOX. The concentrations of pro-inflammatory cytokines were quantified using ELISA, and ultrastructural alterations were evaluated by transmission electron microscopy. To investigate molecular mechanisms in vivo, a PE mouse model was established via daily subcutaneous administration of L-NAME, followed by tail vein delivery of AAV9 carrying shRNA for targeted gene knockdown.

**Results:**

In this study, we demonstrate that BNIP3-mediated mitophagy and NLRP1 inflammasome activation occur in an L-NAME-induced PE mouse model and human PE placenta. The results also indicate that knockdown of BNIP3 abolishes mitophagy and NLRP1 inflammasome activation in JEG3 cells in H/R condition, suggesting a positive regulatory role for the BNIP3 in controlling mitophagy and NLRP1-dependent inflammation. Furthermore, silencing BNIP3 leads to a significant reduction in mitochondrial damage and mtROS production. Treatment with MitoTEMPO after BNIP3 silencing further decreases the expression of NLRP1, while overexpression of NLRP1 nullifies the impact of BNIP3 knockdown. Additionally, knockdown of BNIP3 alleviates placental injury in the PE mouse model.

**Discussion:**

These findings reveal a novel mechanism through which BNIP3-mediated mitophagy exacerbates H/R-induced placental injury by inducing mtROS production and activating the NLRP1 inflammasome in PE.

## 1 Introduction

Preeclampsia (PE), characterized by hypertensive disorders during pregnancy, is a significant contributor to maternal and fetal morbidity and mortality, affecting around 5% to 7% of all pregnant women globally each year (1). Formally defined as the new onset of hypertension accompanied by new-onset proteinuria, PE is often accompanied by other clinical symptoms such as pulmonary edema, liver injury, thrombocytopenia, renal insufficiency, brain dysfunction or visual disturbances (2). Due to the limited understanding of the etiology of PE, there is a scarcity of effective preventive and treatment strategies (3). At the forefront of the maternal-fetal interface, placental insufficiency caused by inadequate remodeling of the maternal vasculature perfusing the intervillous space plays a pivotal role in the development of this syndrome (4). Furthermore, oxidative stress injury in trophoblasts plays a significant role in placental physiology and trophoblast dysfunction (5, 6). Oxidative damage resulting from placental ischemia and hypoxia triggers inflammation and apoptosis (6, 7). Inflammasomes serve as the primary source of inflammatory cytokine release. Nod-like receptor (NLR) family member pyrin domain containing 1 (NLRP1) is an intracellular multimeric protein complex that initiates inflammatory responses through its association with Caspase-1 and ASC (apoptosis-associated speck-like protein containing a CARD) (8).

The NLRP1 inflammasome is an intracellular multimeric protein complex that initiates inflammatory responses and cell death, including pyroptosis and apoptosis, by activating its effector Caspase-1 (9). Numerous studies have demonstrated an upregulation of NLRP1 and Caspase-1 in the placenta of preeclampsia, suggesting that the NLRP1 inflammasome mediates inflammatory responses and contributes to placental injury in preeclampsia (10–12).

Recent studies have demonstrated that mitochondria play a pivotal role in the activation of inflammasomes (13–15). As the primary site of ROS production, mitochondrial homeostasis is intricately linked to inflammasome activation. Mitophagy, a form of selective autophagy, functions to eliminate damaged mitochondria, thus preventing the excessive accumulation of dysfunctional mitochondria, reducing excess ROS, and maintaining normal cellular function.

Mitophagy primarily operates through two mechanisms: the canonical PINK1-PARKIN pathway, which does not involve receptors, and the non-canonical pathway mediated by receptors (16). Under hypoxic conditions, the process is mainly regulated by the interaction between BCL-2 and adenovirus E1B 19-kDa-interacting protein 3 (BNIP3) or its homolog, BNIP3-like (BNIP3L), which directly bind to LC3B to facilitate mitophagy. Notably, BNIP3 has been identified as a crucial component in this process, with HIF-1α serving as an upstream regulator of BNIP3 (17).

Our previous research has demonstrated that excessive BNIP3-mediated mitophagy can induce apoptosis of trophoblasts in the placenta in cases of preeclampsia (PE) (18). While studies have suggested a connection between mitophagy and inflammation triggered by the NLRP3 inflammasome (13), the impact of BNIP3-driven mitophagy on the activation of the NLRP1 inflammasome remains unclear. Therefore, this study aims to clarify the role of BNIP3 in mitophagy and its influence on the activation of the NLRP1 inflammasome in PE.

## 2 Materials and methods

### 2.1 Participants and samples

The study was approved by the Ethical Committee of Shandong Provincial Maternal and Child Health Care Hospital, Affiliated to Qingdao University. Placental tissue samples were collected from 15 pregnant women treated at the hospital between January 2022 and March 2024, all of whom provided informed consent. The definition of preeclampsia (PE) followed guidelines established by the American College of Obstetricians and Gynecologists. In summary, patients exhibited systolic blood pressure exceeding 160 mmHg or diastolic blood pressure exceeding 110 mmHg on at least two occasions, along with significant proteinuria (>2 g per 24 hours or R3+ on dipstick testing in two random urine samples collected at intervals of >4 hours), occurring after 20 weeks of gestation, with no preexisting or chronic hypertension. The sample utilized in this study consists of patients with early-onset PE, defined as the onset of clinical signs occurring before the 34th week (40). Women in the normotensive, normal pregnancy group experienced no complications during pregnancy and delivered healthy neonates at term. The exclusion criteria for the study included transient hypertension during pregnancy, multiple pregnancies, intrauterine fetal death, pregnancies resulting from fertility treatment, fetal chromosomal or congenital abnormalities, gestational diabetes, cardiovascular and immune diseases, and renal disease. Each placental sample was collected within one hour of cesarean section and either snap-frozen in liquid nitrogen for further use or fixed in paraformaldehyde for subsequent paraffin embedding. The clinical characteristics of the enrolled pregnant women are presented in Supporting Information (**Supplementary Table S1**).

### 2.2 Cell culture and treatment

The JEG-3 human choriocarcinoma cell line was obtained from the American Type Culture Collection. These cells were cultured in Dulbecco’s Modified Eagle’s Medium (DMEM) (Hyclone), supplemented with 10% fetal bovine serum (FBS) (GIBCO, New Zealand) and 1% penicillin-streptomycin (Solarbio, China). The cells were maintained in a 5% CO2 incubator at 37°C. Lentiviruses packaging BNIP3 short hairpin RNAs (shRNAs) were synthesized by Shanghai GenePharma Co., Ltd. (Shanghai, China), with the specific shRNA sequence being 5’-GCTAAACCTGAAGAGTGATAT-3’.

### 2.3 Immunoblotting

Total proteins were extracted from the cultured cells or placental tissues using radioimmunoprecipitation assay (RIPA) lysis buffer and quantified with a BCA kit (Solarbio, Beijing, China). Forty micrograms (40 μg) of protein from each sample were separated by sodium dodecyl sulfate-polyacrylamide gel electrophoresis (SDS-PAGE) and subsequently electrotransferred onto polyvinylidene fluoride (PVDF) membranes (Millipore, Buckinghamshire, UK). The membranes were incubated with the specified antibodies, followed by horseradish peroxidase (HRP)-conjugated secondary antibodies. An enhanced chemiluminescence detection kit (Amersham LifeScience, Buckinghamshire, United Kingdom) was employed to visualize the protein bands. The expression of target proteins was quantified by normalization to β-actin levels.

The following antibodies were purchased: LC3A/B(12741) from Cell Signaling Technology (USA), HIF-1 alpha (340462), BNIP3(381756), IL1-bata (516288), TOMM20(R25952), Cleaved- Caspase1(341030) from ZEN-BIOSCIENCE(China), NLRP1 (A16212) from Abclonal (China) β-Actin (6600901) from Proteintech (China), and horseradish peroxidase-labeled goat-anti-mouse immunoglobulin G (GB23301) and horseradish peroxidase-labeled goat-anti-rabbit immunoglobulin G (GB23303) from Servicebio (China).

### 2.4 Immunofluorescence

Paraffin-embedded tissue sections were deparaffinized and rehydrated, followed by antigen retrieval using 0.1% Triton X-100 and sodium citrate. After blocking with 5% goat serum, the slides were incubated with primary antibodies overnight at 4°C in the dark. The primary antibodies used were BNIP3(381756), IL1-bata (516288) (ZEN-BIOSCIENCE, Chengdu, China); IL-18(10663-1-Ap); IL-6 (DF6087, affinity, China); CK7(AB9021, Abcam, USA). Subsequently, the slides were washed and then incubated with a fluorescein-coupled secondary antibody mixture in the dark for 60 minutes at room temperature (1:300 dilution for 1 hour). Nuclei were labeled with DAPI for 10 minutes. The sections were then washed in 1X PBS (3 x 10 minutes), dehydrated in ascending concentrations of ethanol, and sealed with an antifade mounting medium. Visualization was performed using a fluorescence microscope (Olympus, Tokyo, Japan).

### 2.5 Analysis of mitochondrial reactive oxygen species

MitoSOX™ Red Mitochondrial Superoxide Indicator (M36008, Invitrogen) was utilized for the detection of mitochondrial ROS production. Cells were seeded in 96-well plates and then incubated with MitoSOX (5 μM) and Hoechst (5 μl/ml, MedChemExpress, HY-15631) for 1 hour at 37°C, as previously described. Positive staining was observed and the fluorescence intensity was analyzed using the ImageXpress^®^ Micro Confocal System (Molecular Devices, USA).

### 2.6 Transmission electron microscopy

The fresh placenta was dissected into 1 mm³ pieces and then immersed in 2.5% glutaraldehyde for fixation, followed by postfixation with 1% OsO4 for 2 hours. Subsequent steps including dehydration, embedding, polymerization, and lead citrate staining were carried out by a professional service provider (Servicebio, Wuhan, China), following established protocols (18). The embedded samples were sectioned into slices of 60–80 nm thickness and examined using a Hitachi H-7650 transmission electron microscope.

### 2.7 Staining of the mitochondria

Mitochondria, lysosomes, and nuclei were labeled with 50 nM Mito-Tracker Green (Beyotime, C1048), 50 nM Lyso-Tracker Red (Beyotime, C1049), and 5 μg/mL Hoechst (Beyotime, C1027), respectively, following the manufacturer’s instructions. The images were captured using the Confocal Microscopy (Nikon AX, Japan).

### 2.8 Establishment of preeclampsia mouse model

A preeclamptic mouse model was created using a modified method as previously described. This involved 10-week-old ICR mice, both male and female, obtained from Shandong University’s Laboratory Animal Center. The mice were housed in a controlled environment with a 12-hour light/dark cycle at temperatures ranging from 18°C to 22°C, and provided ad libitum access to food and water. Mating was initiated by pairing female mice with male mice at a ratio of 2:1, with the presence of a mating plug designated as gestation day (GD) 0.5.

The mice were divided into two cohorts. In the first cohort consisted of two groups, pregnant mice were randomly allocated into two distinct groups: a control group (Con, n = 6) which was administered 100ul saline solution via subcutaneous injections from GD 9.5 to GD 18.5; an L-NAME group (n = 6) which was administered L-NAME via subcutaneous injections at a dose of 125 mg/kg/day from GD 9.5 to GD 18.5. The relevant data was presented in **Supplementary Figure S1**.

In second cohort, pregnant mice were randomly allocated into four distinct groups: a control group (Con, n = 6) which was injected with 100 μL of the corresponding vector control (AAV9-GFP-vector, n=6) in the tail vein; a BNIP3 knockdown group (sh-BNIP3, n = 6) which was injected with 100 μL of knockdown BNIP3 type 9 adeno-associated virus (AAV9-GFP-sh-BNIP3; synthesized by WZbio); an L-NAME group (L-NAME-Con, n = 6) which was treated with L-NAME and AAV9-GFP-vector; an L-NAME plus BNIP3 knockdown group (L-NAME-sh-BNIP3, n = 6) which was treated with L-NAME and AAV9-GFP-sh-BNIP3. The control groups underwent injections of a saline solution in volumes that were equivalent to those administered to the experimental groups, and these injections were administered via the identical routes and during the corresponding timeframes, ensuring a rigorous and balanced comparison. The maternal systolic and diastolic blood pressures were meticulously recorded on GD 14.5 and 17.5 utilizing tail-cuff plethysmography in conjunction with the advanced BP-2010A Blood Pressure Analysis System. Subsequently, on GD 17.5, comprehensive 24-hour urine samples were gathered for the quantification of protein levels, employing the precise BCA assay. The outcomes of these assessments are visually presented in **Supplementary Figure S2** for detailed analysis. Upon completion of the study period, specifically on GD 18.5, the mice were humanely euthanized through cervical dislocation, adhering to the highest ethical standards. Following this, vital data including placental weights, the total number of fetuses, and individual fetal weights were meticulously compiled and documented. The procured placental tissues were then carefully preserved for subsequent scientific investigations; some were stored at -80°C for molecular and biochemical analyses, while others were fixed in 4% paraformaldehyde for histological and immunohistochemical studies. The protocol for these animal experiments received approval from the Ethical Committee of the Maternal and Child Health Care Hospital of Shandong Province, associated with Qingdao University.

### 2.9 Enzyme-linked immunosorbent assay

The concentration of the pro-inflammatory cytokine IL-1β within the cell culture supernatant was quantitatively assessed employing the Human IL-1β Enzyme-Linked Immunosorbent Assay (ELISA) Kit (EK0392, BOSTER, China), adhering strictly to the manufacturer’s recommended protocol. Similarly, for the evaluation of IL-6 levels, the IL-6 ELISA Kit (EK0411, BOSTER, China) was utilized following the same rigorous methodological guidelines.

### 2.10 Statistical analysis

Statistical evaluations were conducted utilizing the latest GraphPad Prism software, version 9.0, from GraphPad Software in La Jolla, California. For qualitative datasets, including immunoblots and photographic imagery, a minimum of three independent experimental replications were analyzed and presented as mean values, accompanied by the standard deviation of the mean (SD), to ensure reliability and precision. To discern significant differences between two groups, the Student’s t-test was strategically employed. When assessing variations among three or more groups, a comprehensive approach involving one-way ANOVA was utilized, followed by Tukey’s multiple comparisons test. A statistically significant difference was defined as the follows: *p<0.05, **p<0.01, ***p<0.001, **** p<0.0001.

## 3 Results

### 3.1 BNIP3-mediated mitophagy and NLRP1 inflammasome were induced on the placenta of PE

**Figure 1:**
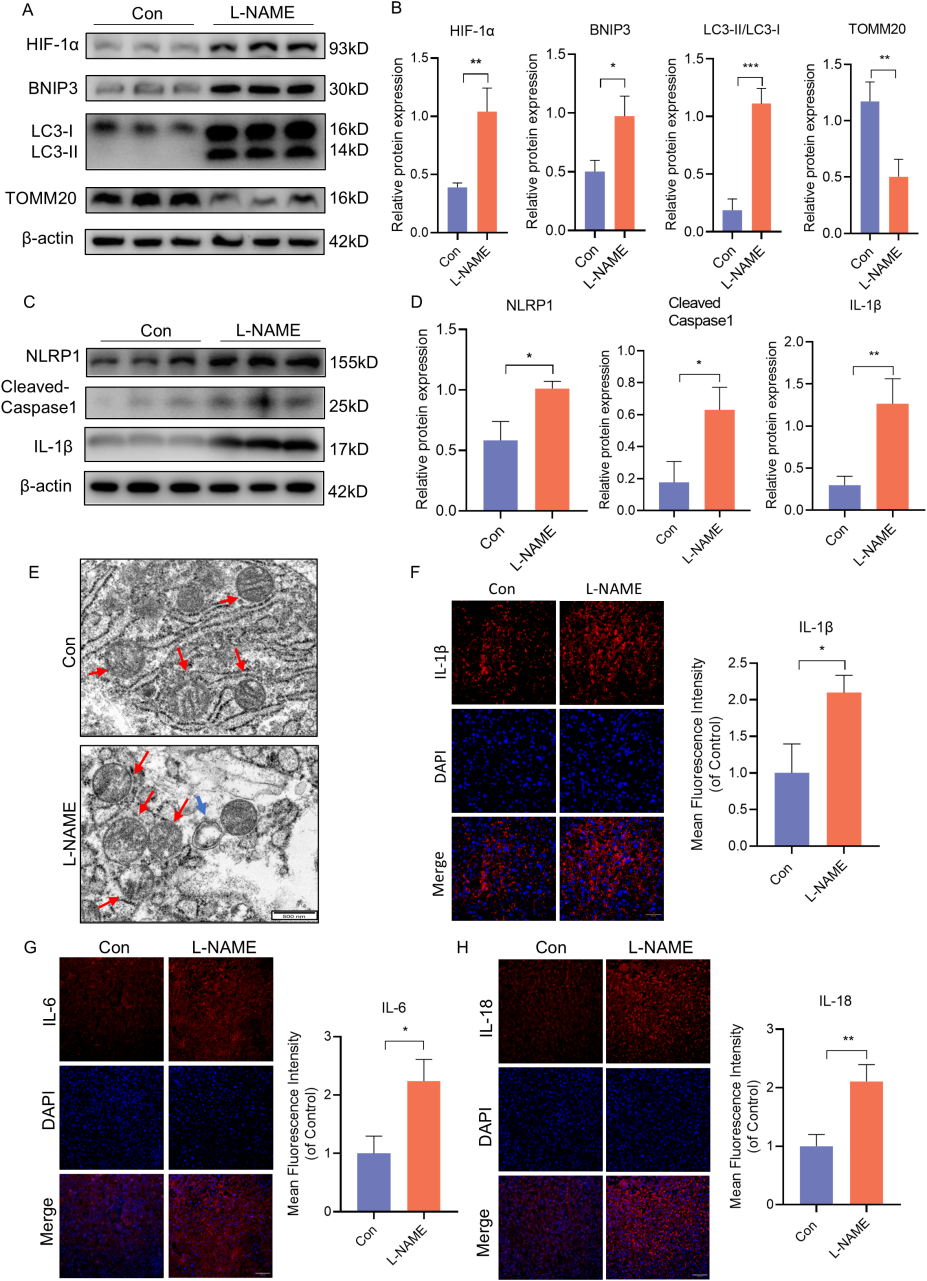
BNIP3 mediated mitophagy and NLRP1 inflammasome were activated in PE-like mice. ICR mice were injected with L-NAME or vehicle. **(A, B)** Results of WB and quantification showing the expression of HIF-1α, BNIP3, the ratio of LC3II/LC3Iand TOMM20 that in the placenta of PE-like mice. **(C, D)** Results and quantification of WB showing the expression of NLRP1, Cleaved-Caspase1 and IL-1β that in the placenta of PE-like mice. **(E)** Representative images of placenta in different groups by transmission electron microscopy. The red arrow indicated mitochondria, the blue triangle indicated autophagosome. Scale bar: 500nm. **(F-H)** Representative images of immunofluorescence labelling IL-1β, IL-6 and IL-18 in placenta of Con and L-NAME groups. The data are shown as mean ± SD and analyzed by Student’s t-test based on at least three independent experiments. *p < 0.05, **p < 0.01, ***p < 0.001.

Our previous research has shown that excessive activation of mitophagy plays a significant role in causing placental damage in preeclampsia (PE). In order to further investigate the relationship between mitophagy and inflammation, we established a PE-like mouse model through L-NAME injection. Compared to the control group, the expression of HIF-1α, BNIP3, and the ratio of LC3II to LC3I was significantly increased, while the expression of TOMM20 was decreased in the PE-like mouse model (**Figures 1A, B**). The protein levels of NLRP1, Cleaved-Caspase-1, and IL-1β are indicative of NLRP1 inflammasome activation. Western blot analysis revealed a notable increase in the expression of NLRP1, Cleaved-Caspase-1, and IL-1β in the L-NAME group (**Figures 1C, D**). Additionally, electron microscopy showed mitochondrial ultrastructural changes with swollen mitochondria and cracked cristae accompanied by autophagosome formation in the L-NAME group (**Figure 1E**). Immunofluorescence staining of placental tissue demonstrated an upregulation in proinflammatory cytokines such as IL-1β, IL-6 and IL-18 in the PE-like group (**Figures 1F-H**). These findings indicate that both mitophagy and NLRP1 inflammation are activated within the placenta of PE mice.

**Figure 2:**
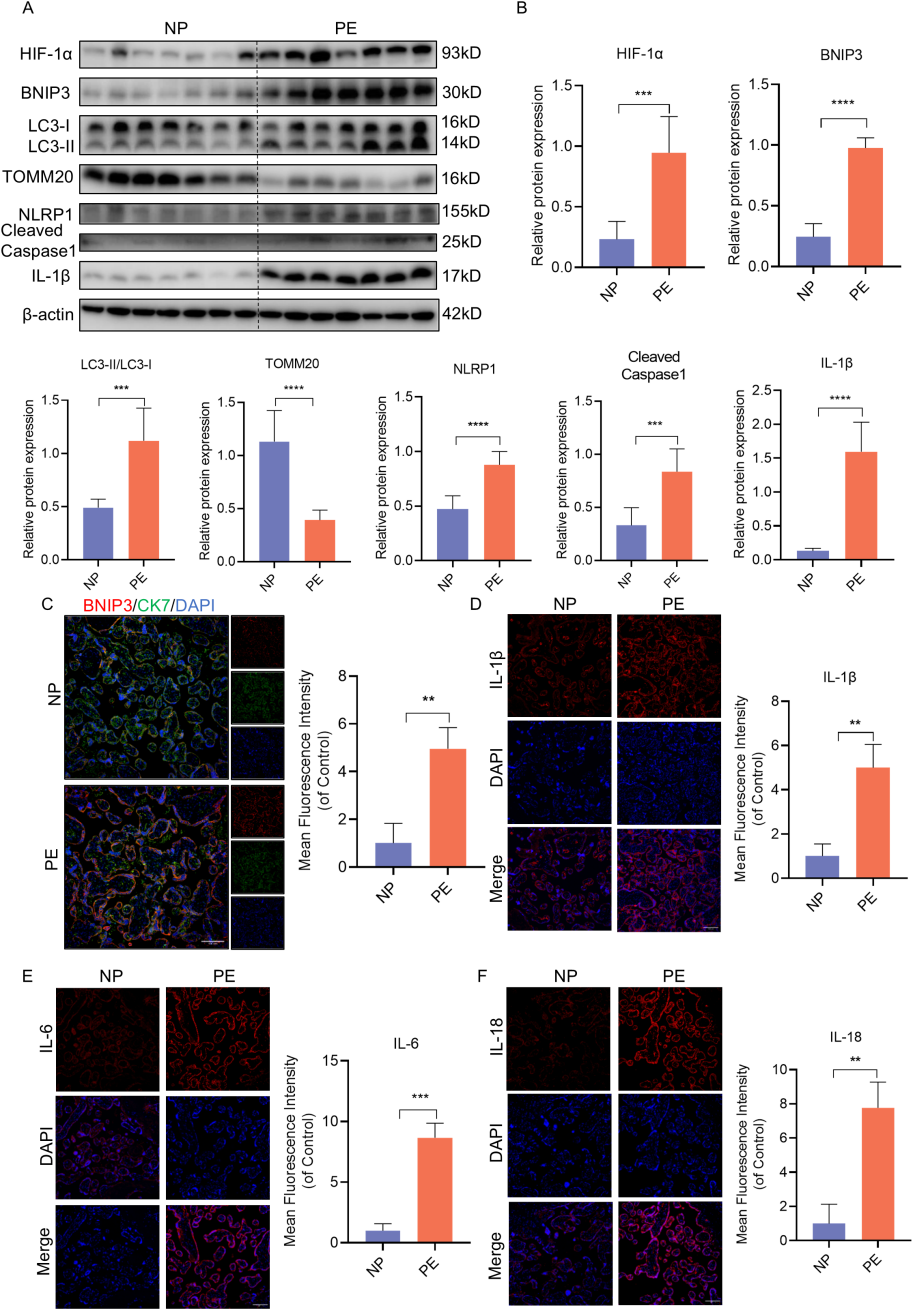
HIF-1α-BNIP3-dependent mitophagy and activation of the NLRP1 inflammasome were associated with PE. **(A, B)** WB analysis and quantification showing the expression of HIF-1α, BNIP3, the ratio of LC3II/LC3I, TOMM20, NLRP1, Cleaved-caspase1, IL-1β that in the villi of healthy pregnant women and pregnant women with PE. **(C)** Representative images of immunofluorescence double-labelling CK-7 and BNIP3 of mice in Con and L-NAME group. **(D–F)** Representative images of immunofluorescence labelling IL-1β, IL-6 and IL-18 in placenta of Con and L-NAME groups. The data are shown as mean ± SD and analyzed by Student’s t-test based on at least three independent experiments. **p < 0.01, ***p < 0.001, ****p < 0.0001.

To validate these findings, we analyzed placenta samples from normal pregnant women (NP) and women with PE. Consistent with the mouse model, the expression of HIF-1α, BNIP3, and the ratio of LC3II to LC3I were significantly increased in PE placentas compared to NP samples, while the expression of TOMM20 was decreased (**Figures 2A, B**). Similarly, immunofluorescence analysis revealed elevated BNIP3 expression in trophoblasts through costaining of BNIP3 and CK7 in PE placentas (**Figure 2C**). The heightened expression of NLRP1, Cleaved-Caspase-1, and IL-1β correlated with NLRP1 inflammasome activation as shown by western blot analysis (**Figures 2A, B**). Furthermore, immunofluorescence analysis demonstrated increased levels of IL-1β, IL-6 and IL-18 to assess proinflammatory cytokine expression in PE placentas (**Figures 2D-F**). These results confirm that mitophagy is activated in PE placentas *in vivo* along with NLRP1 inflammation activation.

### 3.2 Hypoxia-induced activation of BNIP3-mediated mitophagy and NLRP1 inflammasome in trophoblast cells

**Figure 3:**
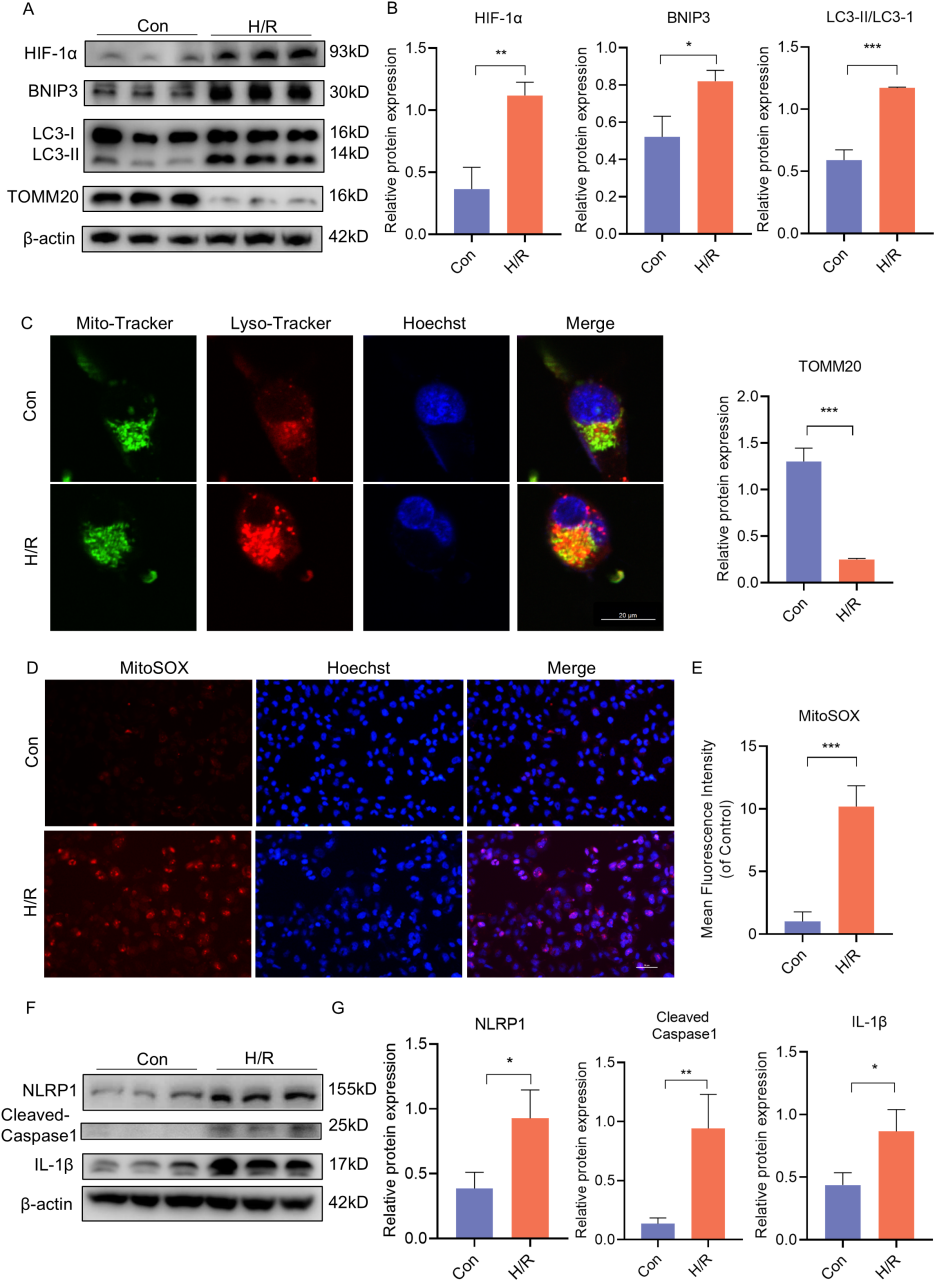
H/R activated BNIP3-mediated mitophagy, the ROS production and the NLRP1 inflammasome in JEG3 cells. JEG3 was used with a hypoxia and reoxygenation (H/R) stimulus to mimic PE-like injury *in vitro*. **(A, B)** Western blotting and corresponding semiquantification were performed to analyze the expression of HIF-1α, BNIP3, the ratio of LC3II/LC3I, TOMM20 in JEG3 cells treated in H/R condition. **(C)** Representative images of fluorescence double labelling mitochondrial (Mito-Tracker) and lysosome (Lyso-Tracker) marker. Scale bar: 20 μm. **(D, E)** MitoSOX was used to detect the mitochondrial ROS and analyzed by confocal microscopy (n = 3). Scale bar, 50 μm. **(F, G)** Western blotting and corresponding semiquantification were performed to analyze the expression of NLRP1, Cleaved-Caspase1 and IL-1β in JEG3 cells treated in H/R condition. The data are shown as mean ± SD and analyzed by Student’s t-test based on at least three independent experiments. *p < 0.05, **p < 0.01, ***p < 0.001.

Following this, JEG3 cells were subjected to H/R conditions *in vitro* to simulate the hypoxic environment in PE placenta. In order to investigate the activation of BNIP3-dependent mitophagy induced by hypoxia, JEG3 cells were cultured under H/R conditions. Western blot analysis revealed that H/R treatment led to a decrease in TOMM20 expression and an increase in HIF-1α and BNIP3 expression, as well as an elevation in the ratio of LC3II/LC3I within the group (**Figures 3A, B**). Additionally, Mito-Tracker and Lyso-Tracker were used to label mitochondria and lysosomes respectively. It was observed that JEG3 cells treated with H/R exhibited a significant increase in co-localization of Mito-Tracker Green and Lyso-Tracker Red, indicating a notable enhancement in mitophagy following H/R treatment (**Figure 3C**). Subsequently, mitochondrial function was assessed by quantifying mitochondrial ROS (mtROS) production using MitoSOX, a fluorogenic dye specifically targeting mitochondria for measuring their superoxide anion generation. Analysis of fluorescence intensity demonstrated significantly higher mtROS generation in the H/R group compared to the control (**Figures 3D, E**). Furthermore, H/R-treated JEG3 cells also resulted in activation of the NLRP1 pathway as evidenced by elevated protein expression of NLRP1, Cleaved-Caspase1, and IL-1β (**Figures 3F, G**). These findings indicate that not only mitophagy but also NLRP1-mediated inflammation was activated in response to H/R treatment.

### 3.3 BNIP3 deficiency reduced mitophagy, mitochondrial ROS production, and the induction of NLRP1 inflammasome activation in H/R-induced trophoblasts

**Figure 4:**
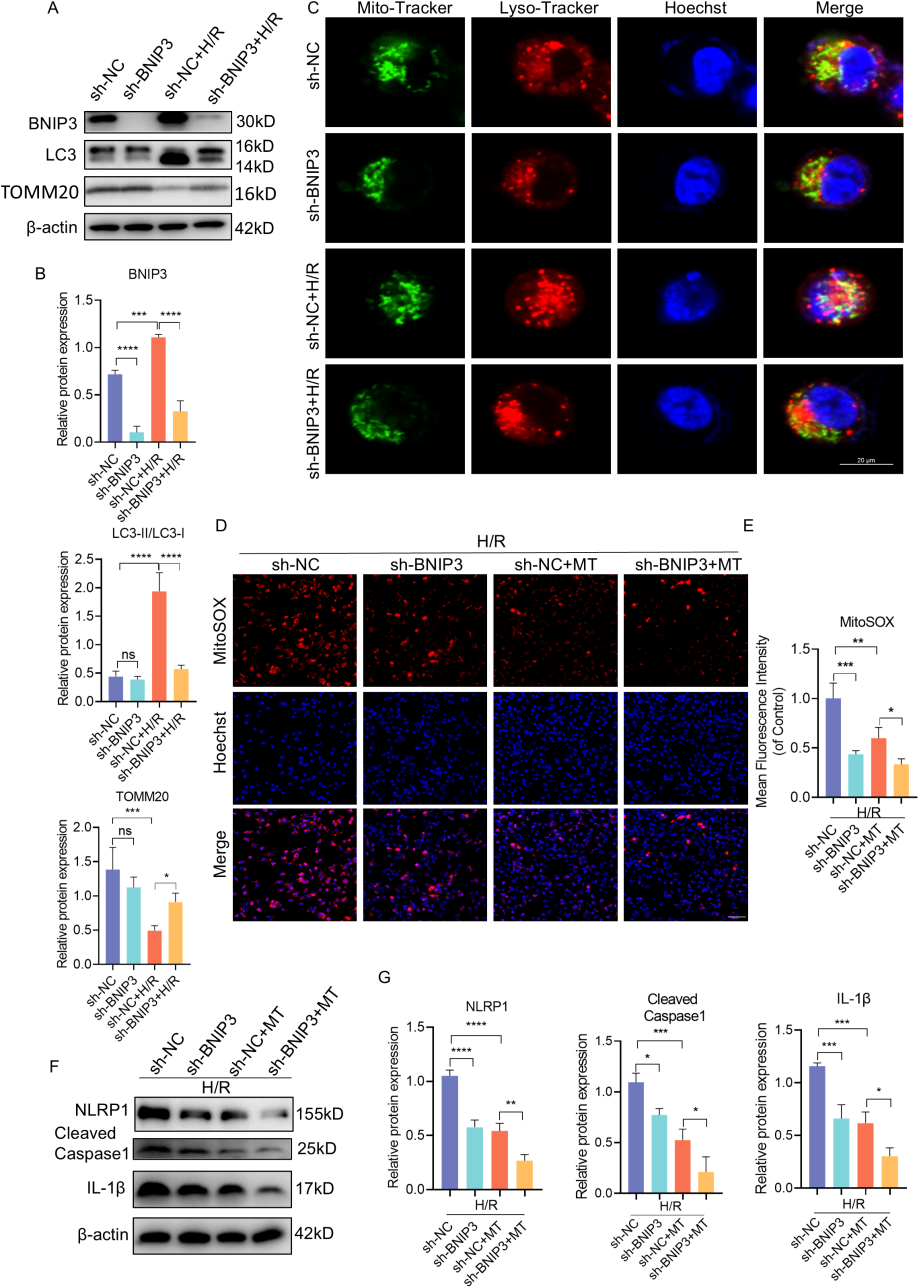
BNIP3 silencing alleviated mitophagy, ROS production and NLRP1 inflammasome activation. ShRNA against BNIP3 and sh-NC were transfected into JEG3 cells. **(A, B)** The alteration of BNIP3, LC3II/LC3Iand TOMM20 was detected by Western blotting. **(C)** Representative images of immunofluorescence double labelling mitochondrial (Mito-Tracker) and lysosome (Lyso-Tracker) marker. Scale bar: 20 μm. **(D, E)** MitoSOX was used to detect the mitochondrial ROS and analyzed by confocal microscopy (n = 3). Scale bar, 100 μm. **(F, G)** Western blotting and corresponding semiquantification were performed to analyze the expression of NLRP1, Cleaved-Caspase1 and IL-1β. The data are shown as mean ± SD and analyzed by one-way ANOVA test followed by Tukey–Kramer multiple comparison test based on at least three independent experiments. *p < 0.05, **p < 0.01, ***p < 0.001, ****p < 0.0001. ns: not significant.

To further elucidate the impact of BNIP3 on H/R injury, our focus was on mitophagy. We utilized shRNA to knock down BNIP3 in JEG3 cells and investigate its role in H/R-induced JEG3 cells. After silencing the BNIP3 gene, JEG3 cells were exposed to hypoxia for 24 hours. The successful inhibition of BNIP3 expression by shRNA transfection was confirmed through immunoblot analysis. Western blot analysis showed a significant decrease in BNIP3 protein expression levels in the sh-BNIP3 and sh-BNIP3+H/R groups (**Figures 4A, B**). Furthermore, colocalization of Mito-Tracker and Lyso-Tracker demonstrated a reduction in mitophagosome formation after BNIP3 knockdown (**Figure 4C**). Interestingly, H/R injury led to excessive ROS production in JEG3 cells as evidenced by MitoSOX staining; however, this effect was reduced upon BNIP3 knockdown (**Figures 4D, E**). These findings suggest that under H/R conditions, BNIP3-mediated mitophagy and mitochondrial ROS production are activated but suppressed when BNIP3 is silenced.

In order to explore the influence of BNIP3 and mtROS on NLRP1 inflammasome activation, we treated the cells with Mito-Tempo (MT) alongside intervention targeting BNIP3. Both interventions resulted in decreased NLRP1, Cleaved-Caspase1, and IL-1β expression (**Figures 4F, G**), indicating that both BNlP3-mediated mitophagy and mtROS play a role in regulating NLRP1 inflammasome activation.

### 3.4 Activation of the NLRP1 inflammasome partially attenuates the protective effect of BNIP3 silencing against hypoxia injury in JEG3 cells

**Figure 5:**
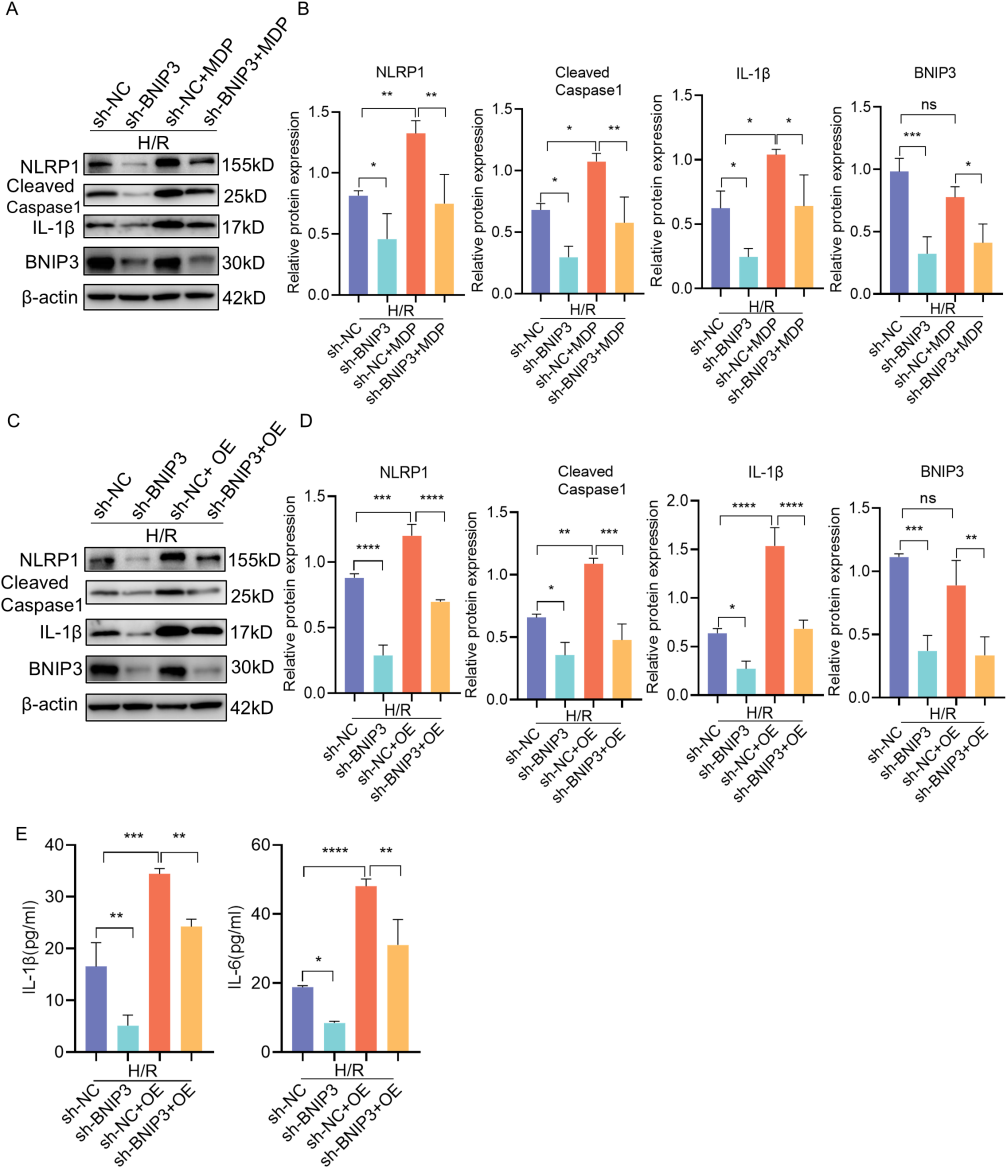
BNIP3 mediated NLRP1 inflammasome activation induced by H/R. **(A, B)** JEG3 cells knockdown BNIP3 were treated with MDP (NLRP1 targeted activator), Western blotting and corresponding semiquantification were performed to analyze the expression of NLRP1, Cleaved-Caspase1, IL-1β and BNIP3. **(C, D)** JEG3 cells were transfected with both shRNA targeted BNIP3 and NLRP1 overexpression plasmid. Western blotting and corresponding semiquantification were performed to analyze the expression of NLRP1, Cleaved-Caspase1, IL-1β and BNIP3. **(E)** ELISA assay was used to detected the IL-1β and IL-6 expression in supernant of cell in the four groups. The data are shown as mean ± SD and analyzed by one-way ANOVA test followed by Tukey–Kramer multiple comparison test based on at least three independent experiments. *p < 0.05, **p < 0.01, ***p < 0.001, ****p < 0.0001. ns: not significant.

To further investigate the relationship between BNIP3-mediated mitophagy and the NLRP1 inflammasome, JEG3 cells were treated with MDP (NLRP1 targeted activator) after BNIP3 knockdown to induce NLRP1 inflammasome activation prior to exposure to H/R conditions (**Figures 5A, B**). The reduction in NLRP1 inflammasome activation observed with BNIP3 silencing was reversed by MDP treatment, as demonstrated by Western blot analysis. To validate the regulatory role of BNIP3 in NLRP1 inflammasome activation, NLRP1 was overexpressed using a plasmid. Consistent with MDP treatment, the overexpression of NLRP1 counteracted the inhibitory effect of BNIP3 on NLRP1 inflammasome activation (**Figures 5C, D**). Additionally, ELISA assays were used to analyze and compare the levels of IL-1β and IL-6 in the cell supernatant among the four groups, confirming a decrease in inflammatory factors in the sh-BNIP3 group. Furthermore, overexpression of NLRP1 nullified the impact of BNIP3 knockdown (**Figure 5E**). These findings suggest that BNIP3-mediated mitophagy induces NLRP1 inflammasome activation.

### 3.5 Deficiency of BNIP3 resulted in decreased mitophagy, reduced activation of the NLRP1 inflammasome, and dysfunction of the placenta with PE

**Figure 6:**
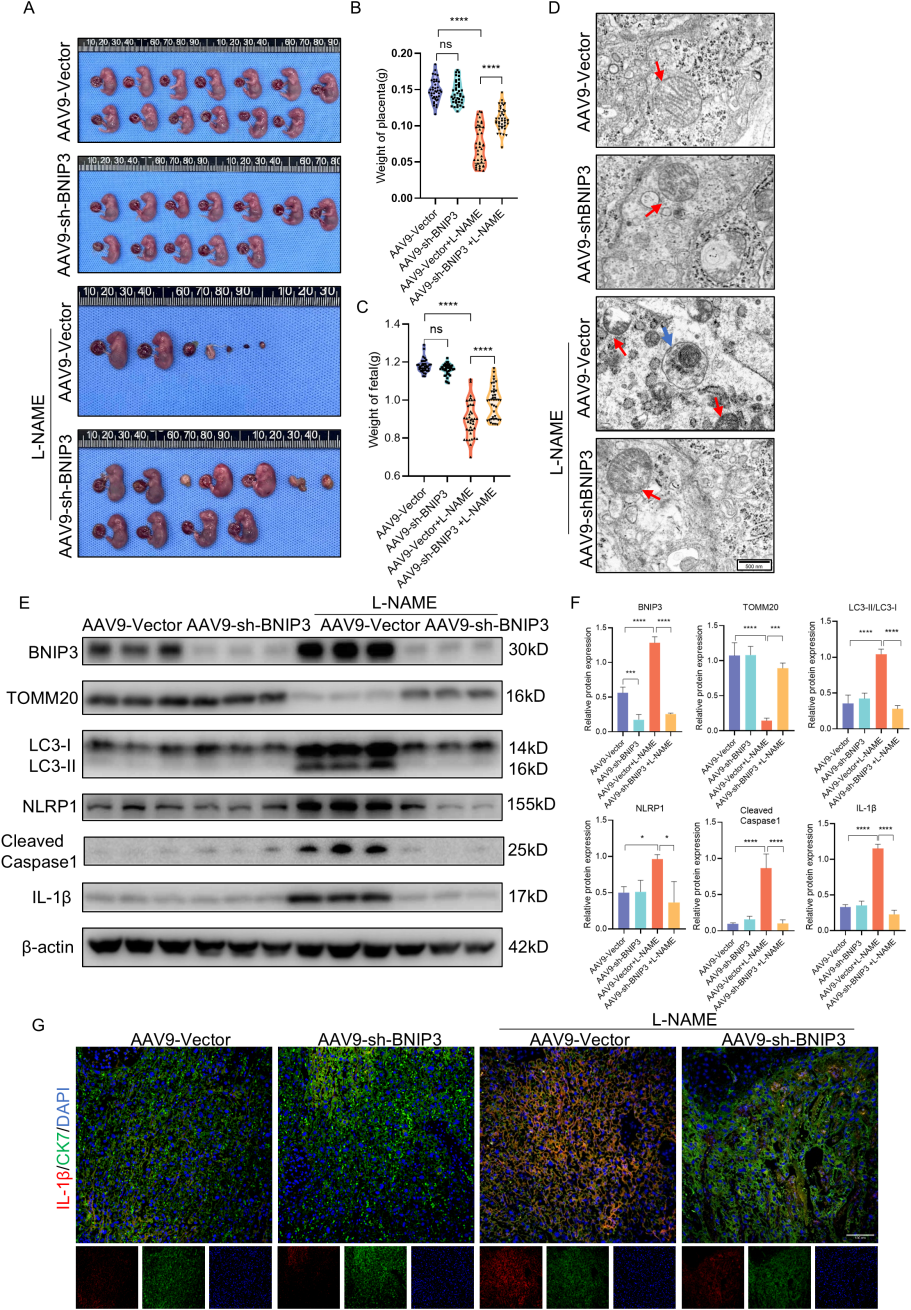
BNIP3 deficiency blocked NLRP1 inflammasome activation and mitigated placental injury of PE. **(A-C)** Representative images of the gestational day (GD)18.5 fetuses of the AAV9-Vector group (n = 6) and AAV9-shBNIP3 group (n = 6), L-NAME+ AAV9-Vector group (n = 6) and L-NAME+AAV9-shBNIP3 group (n = 6), and statistics of weight of placentas and fetuses. **(D)** Representative images of placenta in different groups by transmission electron microscopy. **(E, F)** Western blotting and corresponding semiquantification were performed to analyze the expression of BNIP3, TOMM20, the ratio of LC3II/LC3I, NLRP1, Cleaved-Caspase1 and IL-1β. **(G)** Representative images of immunofluorescence labelling IL-1β and CK7 in placentas of different groups. The data are shown as mean ± SD and analyzed by one-way ANOVA test followed by Tukey–Kramer multiple comparison test based on at least three independent experiments. *p < 0.05, ***p < 0.001, ****p < 0.0001. ns: not significant.

**Figure 7:**
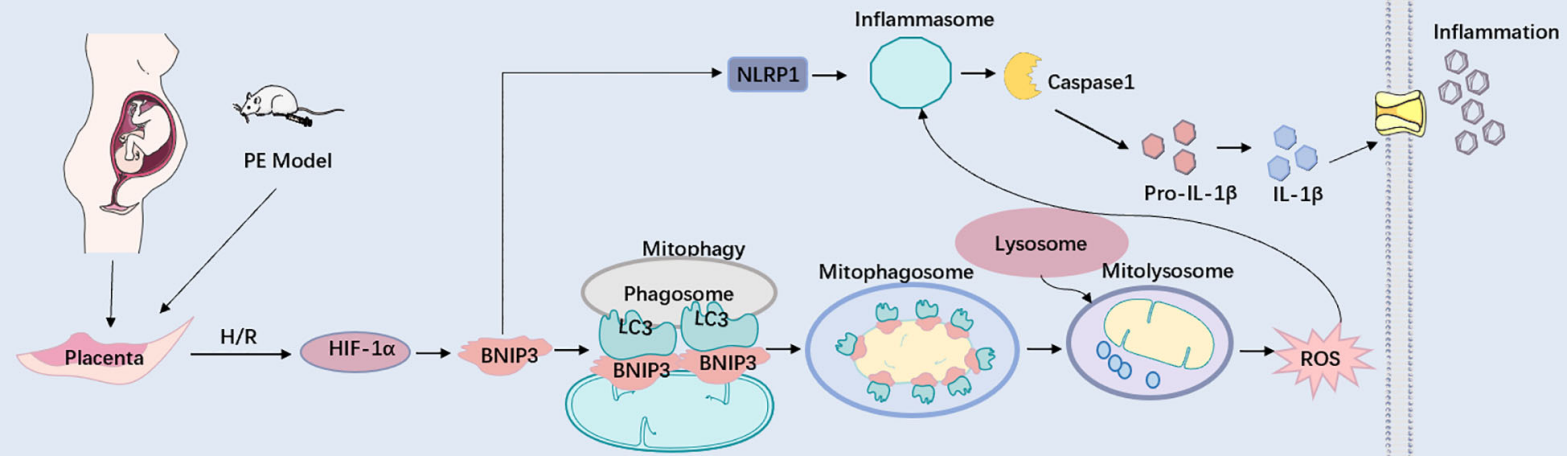
BNIP3 upregulation in trophoblasts aggravates placental injury of PE via activating mitophagy and NLRP1-inflammasome. Working model depicting the BNIP3-mediated mitophagy and NLRP1-associated inflammation in trophoblasts during PE. Under pathologically H/R conditions that mimic PE, BNIP3 is activated, subsequently enhancing downstream mitophagy and ROS production. Moreover, BNIP3 promotes the activation of NLRP1, resulting in an increased release of inflammatory mediators and exacerbating placental damage associated with PE both *in vivo* and *in vitro*. Under pathologically H/R conditions that replicate PE, BNIP3 is activated, which subsequently enhances downstream mitophagy and ROS production. Furthermore, BNIP3 facilitates the activation of NLRP1, leading to an increased release of Inflammatory factors and aggravating placental damage of PE *in vivo* and *in vitro*.

The knockdown of BNIP3 using AAV9-sh-BNIP3 adeno-associated virus in a PE-like mouse model was employed to validate our hypothesis regarding the role of increased BNIP3 levels and its contribution to PE. We observed that the weight of placentas and fetuses in the L-NAME group was significantly lower than that in the control group (**Figures 6A-C**). Conversely, downregulation of BNIP3 reversed this trend, leading to a rebound in the weight of placentas and fetuses in the L-NAME+AAV9-sh-BNIP3 group. Furthermore, transmission electron microscopy (TEM) images revealed an improvement in mitochondrial ultrastructure as a result of BNIP3 downregulation. The stage of mitophagy, specifically mitophagosome formation, was identified in the placenta of the L-NAME group (**Figure 6D**). To confirm the inhibitory effect of AAV9-sh-BNIP3, we analyzed BNIP3 levels in the placentas from four groups of pregnant mice (**Figures 6E, F**). Our findings demonstrated a significantly lower expression of BNIP3 in the AAV9-sh-BNIP3 groups compared to the AAV9-vector groups. Western blot analysis also revealed an increase in TOMM20 expression in the L-NAME+AAV-sh-BNIP3 group compared to the L-NAME group. Additionally, the ratio of LC3II to LC3I was significantly reduced in the L-NAME+AAV-sh-BNIP3 placenta (**Figures 6E, F**). We assessed the levels of NLRP1, Cleaved-Caspase-1, and IL-1β in the placentas of these four groups. The results indicated that knockdown BNIP3 with AAV9-sh-BNIP3 counteracted the inductive effect of L-NAME on NLRP1 inflammasome activation. Furthermore, immunofluorescence analysis of IL-1β and CK7 was performed to assess proinflammatory cytokine expression in trophoblasts, which were decreased in AAV9-sh-BNIP3+L-NAME placentas (**Figure 6G**). These observations were consistent with findings from JEG3 cells, inhibiting mitophagy by downregulating BNIP3 expression ultimately improved placental function by relieving NLRP1-inflammasome activation in PE. An integrative schematic model delineating the hypothesized signaling pathways was constructed based on our findings (**Figure 7**).

## 4 Discussion

The present study demonstrates the significance of the HIF-1α-BNIP3 pathway in activating the NLRP1 inflammasome under hypoxic conditions, particularly in relation to placental damage caused by hypoxia. Our findings indicate that HIF-1α and BNIP3, as well as its mediated mitophagy, are upregulated in the placentas of pregnant women with preeclampsia (PE), as well as in a PE-like mouse model. Consistent with these results, BNIP3-mediated mitophagy is activated in trophoblasts under hypoxia/reoxygenation (H/R) conditions *in vitro*. Furthermore, we observed activation of the NLRP1 inflammasome pathway and an increase in mitochondrial reactive oxygen species (mtROS) production under these conditions. Additionally, our study reveals that deficiency of BNIP3 rescues both mitophagy activation and NLRP1 inflammasome activation induced by hypoxia both *in vitro* and *in vivo*. These findings provide further evidence for the relationship between BNIP3-mediated mitophagy and the NLRP1 inflammasome, highlighting their role in the development of PE.

The placenta is essential for facilitating communication between the mother and the fetus, ensuring maternal well-being and normal fetal development (19). Various pathological factors, such as hypoxia, inflammation, and metabolic abnormalities, are closely linked to preeclampsia in a multifaceted manner. Within the placenta, trophoblasts subjected to oxidative stress damage due to hypoxia play a pivotal role in the pathogenesis of preeclampsia (4, 6). Additionally, mitochondrial damage characterized by structural breakdown and excessive mitophagy induced by hypoxia is intricately associated with placental dysfunction. Previous research findings, including our own published studies, indicate that mitophagy contributes to placental injury caused by hypoxia in preeclampsia (18, 20). It has been suggested that BNIP3-mediated mitophagy may have a more significant impact than PINK/PARKIN-mediated mitophagy due to its heightened sensitivity to hypoxic environments. Numerous studies have suggested that mitophagy plays a protective role in certain hypoxic tissue injuries. However, our previous research has shown that inhibiting BNIP3-mediated mitophagy reduced trophoblast apoptosis and placenta injury in a PE-like mouse model (18). In this study, we further demonstrate that BNIP3-mediated mitophagy is activated under hypoxic/reoxygenation (H/R) conditions both *in vitro* and in the placenta of PE models. Additionally, the absence of BNIP3 results in decreased accumulation of mitochondrial ROS and inflammatory responses in the placenta of PE-like mice. These conflicting findings indicate that the functions of BNIP3 in mitochondrial regulation may depend on different cellular environments.

Accumulating evidence has demonstrated the critical role of inflammation and oxidative stress, particularly NLRPs, in the pathogenesis of PE (6, 21). The NLRP1 signaling pathway acts as a central component in innate immunity, regulating inflammatory responses and coordinating the host’s defense mechanisms against pathogens (22). This pathway has attracted significant attention due to its pivotal involvement in various inflammatory disorders and infectious diseases. NLRP1 serves as a key sensor for danger signals and microbial components, initiating the assembly of the inflammasome complex, which subsequently activates Caspase-1. This activation leads to the production and release of pro-inflammatory cytokines, specifically interleukin-1β (IL-1β) and interleukin-18 (IL-18) (23). Dysregulation of NLRP1 signaling has been implicated in the pathogenesis of numerous diseases, including autoimmune disorders, metabolic syndromes, and neurodegenerative conditions (24–28). However, its physiological functions and implications in obstetric complications remain unclear. Limited research has been conducted on the impact of hypoxia on NLRP1 activation. However, existing studies have indicated that NLRP1 interacts with HIF-1α under hypoxic conditions in various tissues, such as microvascular endothelium (29), neurons (30), liver (31), and myocardium (32), thereby facilitating ASC-Caspase-1-IL-1β signaling cascades. Nonetheless, other regulatory mechanisms under hypoxic conditions require further investigation. Our study demonstrates that the expression level of NLRP1 is upregulated in human trophoblasts in placenta samples from preeclampsia patients compared to normal pregnant women. This upregulation is associated with hypoxia and its related oxidative stress. Furthermore, our findings suggest that BNIP3-mediated mitophagy plays a significant role in regulating the NLRP1 inflammasome.

Previous research has suggested that mitophagy plays a critical role in the activation of the NLRP3 inflammasome. It has been documented that HIF-1α-BNIP3-mediated mitophagy mitigates renal fibrosis by inhibiting the activation of the NLRP3 inflammasome, and BNIP3-mediated mitophagy also exerts a protective effect on neuroinflammation by suppressing the assembly of the NLRP3 inflammasome (33, 34). The role of BNIP3 in regulating the NLRP1-Caspase1 signaling pathway has not previously been reported. In our current study, we observed that hypoxia-induced activation of BNIP3-mediated mitophagy triggers the NLRP1-Caspase1 pathway. Knockdown of BNIP3 reduced both NLRP1 and Caspase1 expression, as well as inflammatory cytokine release, which was reversed by overexpression of NLRP1. Furthermore, we demonstrated that trophoblast inflammation was attenuated by reducing mitochondrial ROS production with MitoTEMPO, a mitochondria-targeted superoxide dismutase mimetic. Consistent with our findings, several studies have unequivocally shown that ROS directly triggers the activation of NLRP1 in various conditions such as age-related neuronal damage, BAP-induced lung epithelial injury, and H_2_O_2_-induced neuronal damage (35–37). Furthermore, the ablation of BNIP3 ameliorated placental inflammatory injury in PE-like mice. However, multiple studies have demonstrated that mitophagy suppresses inflammation, including NLRP3 inflammasome-mediated pyroptosis in rheumatoid arthritis, aging muscle, and chronic alcohol exposure-induced cognitive impairment (34, 38, 39). These findings appear to contradict our results, possibly due to the diverse interaction patterns between mitophagy and inflammation at different stages. These data indicate that NLRP1 is downstream of the regulatory pathway of BNIP3-mediated mitophagy and the ROS it generates. The hypoxia/reoxygenation condition leads to excessive activation of mitophagy, which significantly contributes to mitochondrial damage and subsequent ROS production, exacerbating their harmful effects. In conclusion, placental hypoxia induces the upregulation of BNIP3, leading to enhanced mitophagy and ROS production. Subsequently, this activates NLRP1-inflammasome production, resulting in the release of inflammatory factors and subsequent placental inflammatory damage. The study further elucidates the role of BNIP3 in inflammation in preeclampsia (PE) and proposes that BNIP3 could potentially serve as a target for the treatment of pregnancy-related diseases manifesting as placental inflammation, such as PE.

## Data Availability

The original contributions presented in the study are included in the article/**Supplementary Material**. Further inquiries can be directed to the corresponding authors.
