# Supplementary material for: BNIP3-mediated mitophagy aggravates placental injury in preeclampsia via NLRP1 inflammasome
Source: Front Immunol. 2025 Apr 2;16:1530015. doi: 10.3389/fimmu.2025.1530015 (PMC11999839; doi:10.3389/fimmu.2025.1530015)
Supplement: Supplementary file 1 [file DataSheet1.docx]

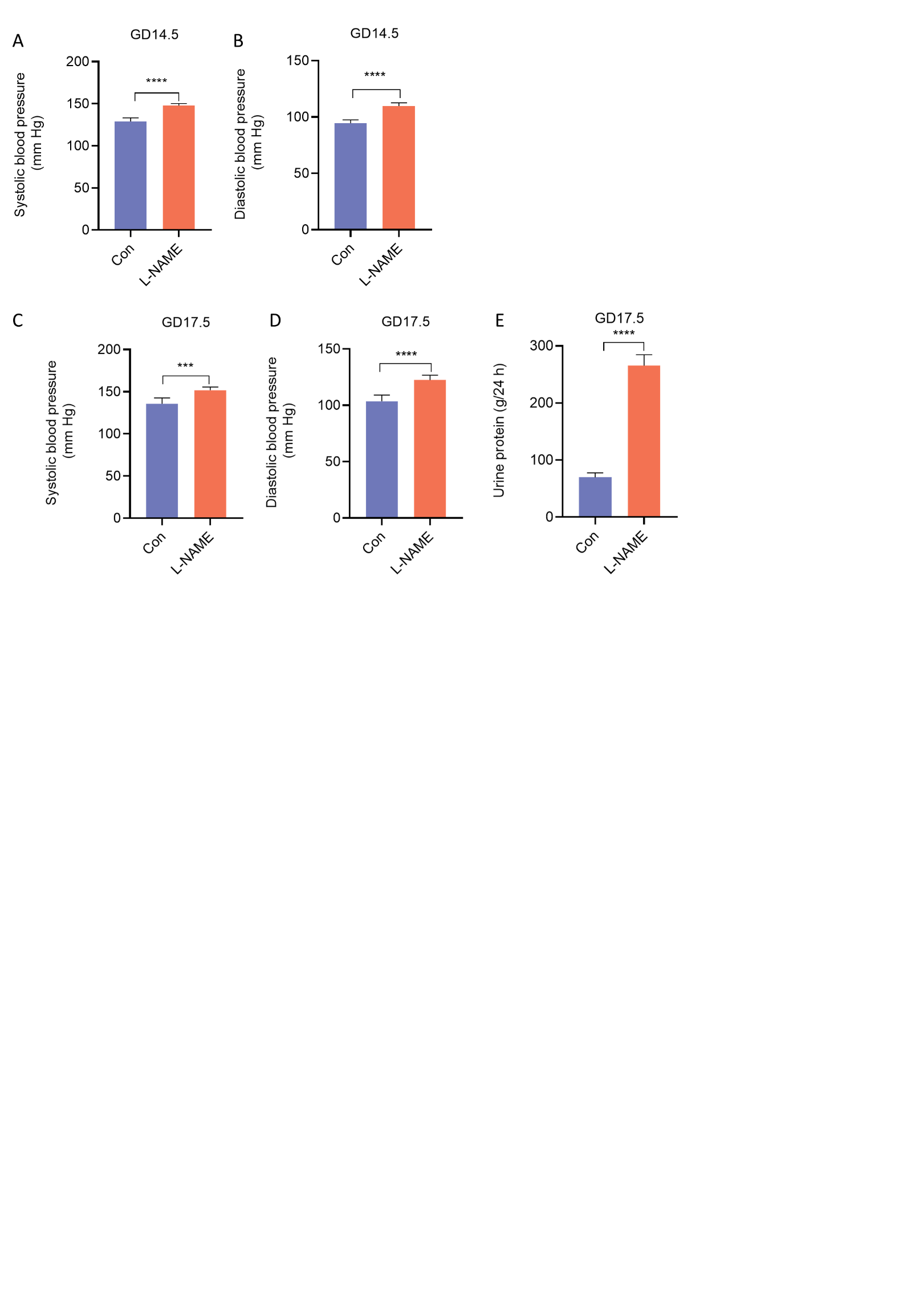


Supplementary Fig. 1. **Blood pressure and urine protein of mice in the first cohort.** systolic blood pressure (mmHg) (A) and diastolic blood pressure (mmHg) (B) of mice in each group (n=6) on GD14.5. systolic blood pressure (mmHg) (C), diastolic blood pressure (mmHg) (D)and urine protein of mice in each group on GD17.5(E). The data are shown as mean ± SD and analyzed by Student’s t test based on at least three independent experiments. ns, no significance , *p < 0.05, **p < 0.01，***p < 0.001，****p < 0.0001.


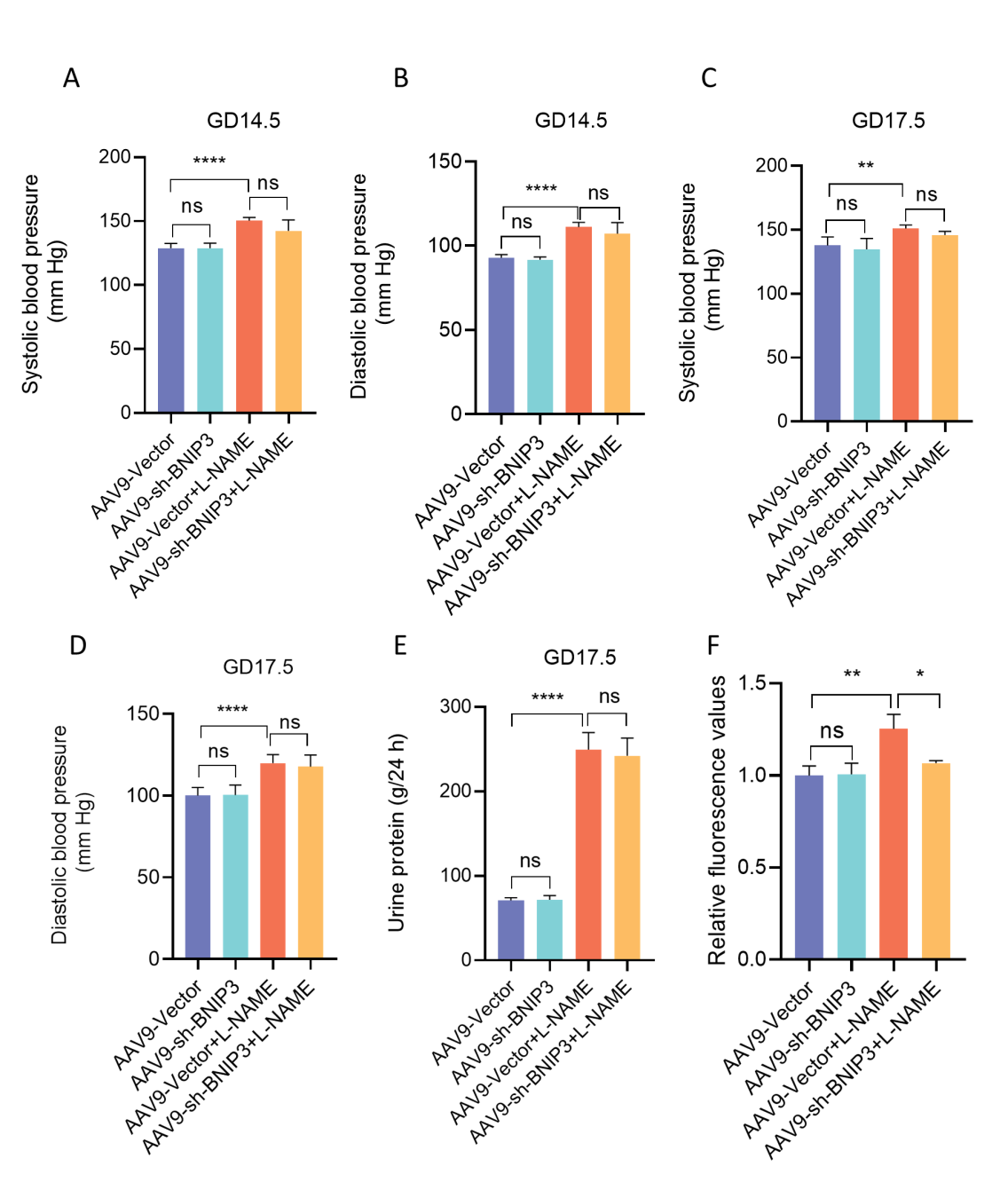


Supplementary Fig. 2. **Effect of AAV9-shBNIP3 administration on blood pressure and urine protein in**

**PE-like mouse model.** systolic blood pressure (mmHg) (**A**) and diastolic blood pressure (mmHg) (**B**) of mice in each group (n=6) on GD14.5. systolic blood pressure (mmHg) (**C**), diastolic blood pressure (mmHg) (**D**)and urine protein of mice in each group on GD17.5(**E**); The ROS level of placenta tissues in each group on GD17.5(**F**). The data are shown as mean ± SD and analyzed by one-way ANOVA test followed by Tukey–Kramer multiple comparison test based on at least three independent experiments. ns, no significance , *p < 0.05, **p < 0.01，***p < 0.001，****p < 0.0001.


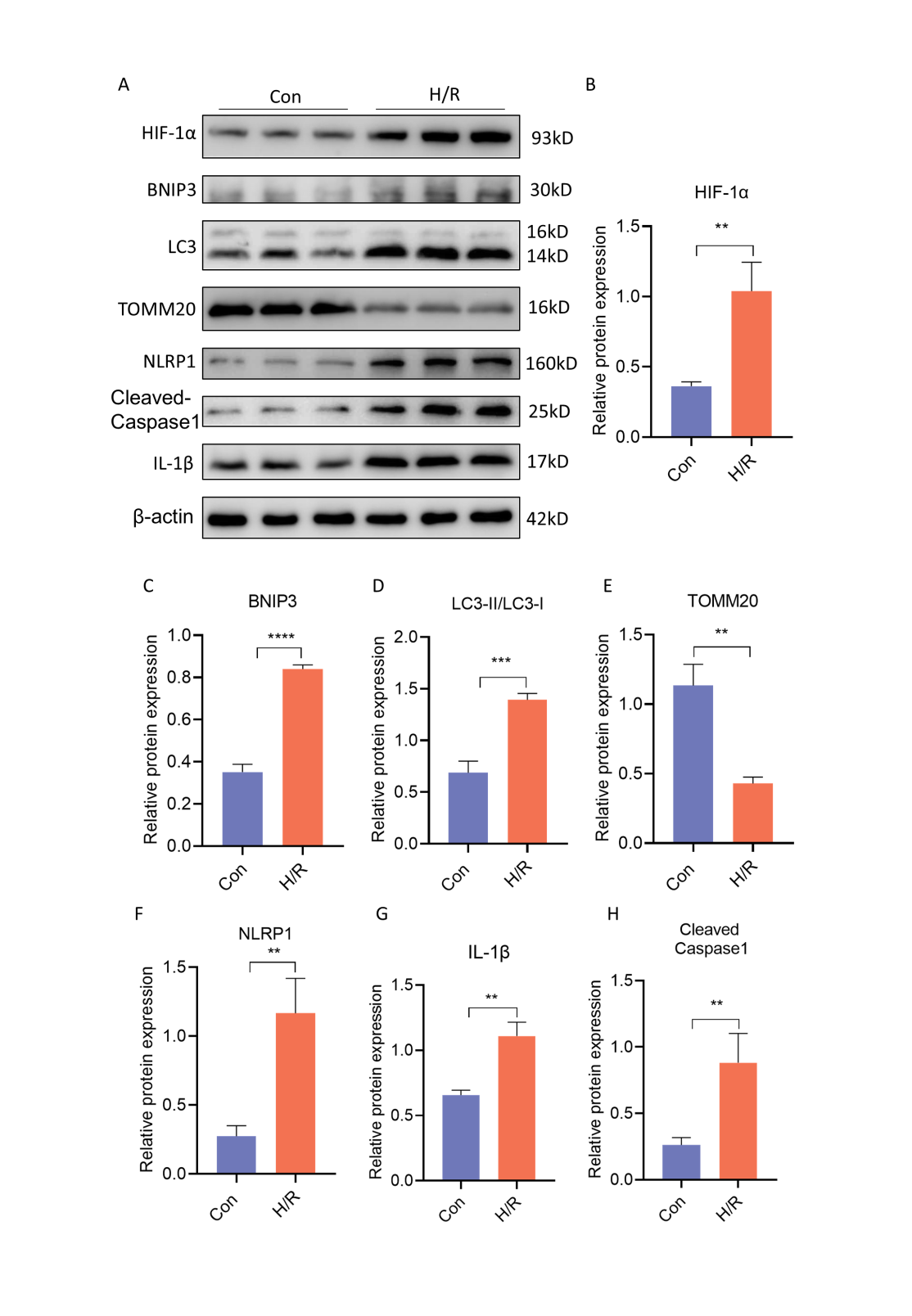


Supplementary Fig. 3. **H/R activated BNIP3-mediated mitophagy and NLRP1 in HTR-8/Svneo cell.** Western blotting(A) and corresponding semiquantification(B-H) were performed to analyze the expression of HIF-1α, BNIP3, the ratio of LC3Ⅱ/LC3Ⅰ, TOMM20, NLRP1, Cleaved-Caspase1 and IL-1β in HTR-8/Svneo cells treated in H/R condition. The data are shown as mean ± SD and analyzed by Student's t-test based on at least three independent experiments. *p < 0.05, **p < 0.01, ***p < 0.001, ****p < 0.0001.
