# Supplementary material for: BNIP3-mediated mitophagy aggravates placental injury in preeclampsia via NLRP1 inflammasome
Source: Front Immunol. 2025 Apr 2;16:1530015. doi: 10.3389/fimmu.2025.1530015 (PMC11999839; doi:10.3389/fimmu.2025.1530015)
Supplement: Supplementary file 2 [file DataSheet2.pdf]

Figure1

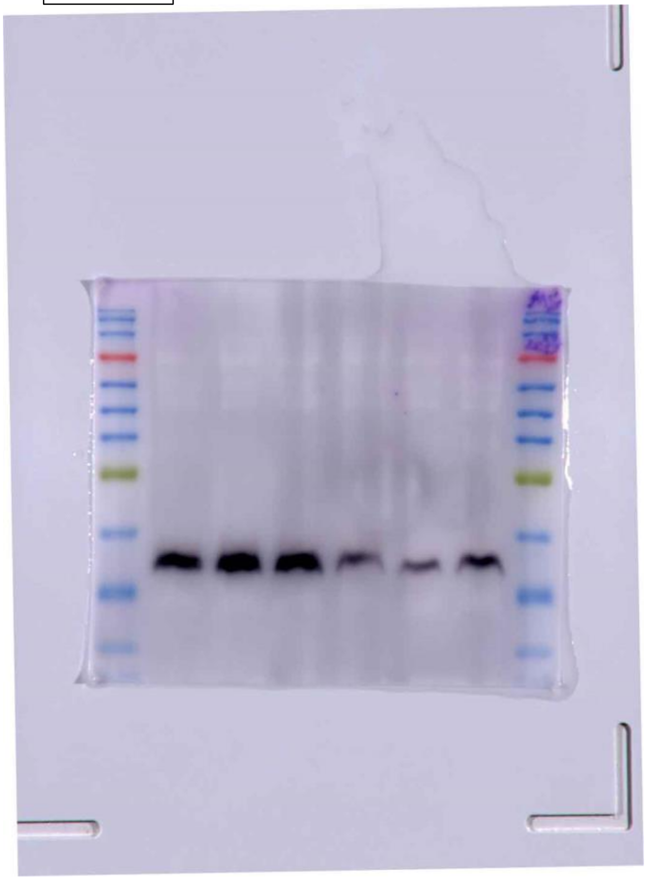

TOMM20

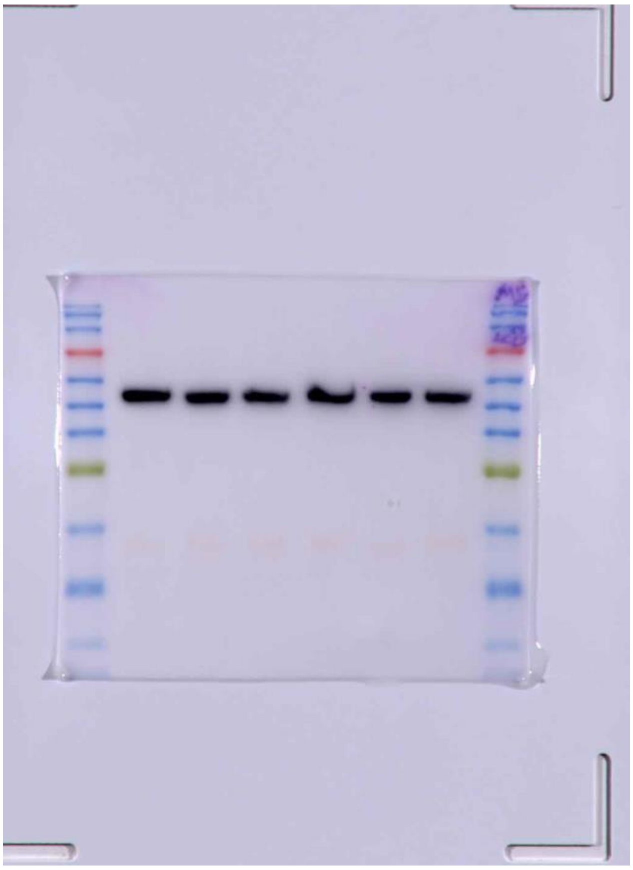

β-actin

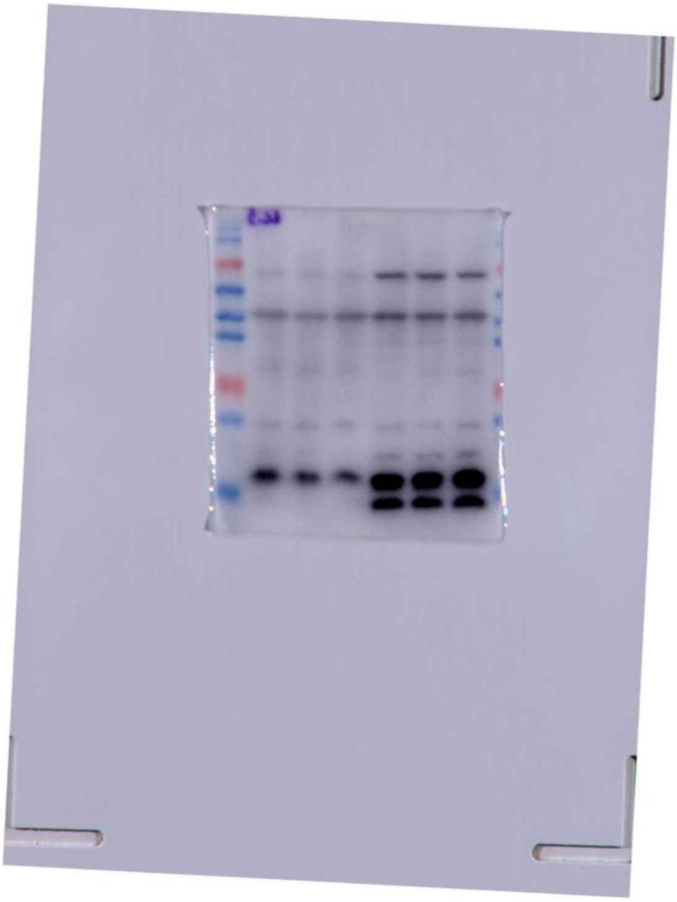

LC3

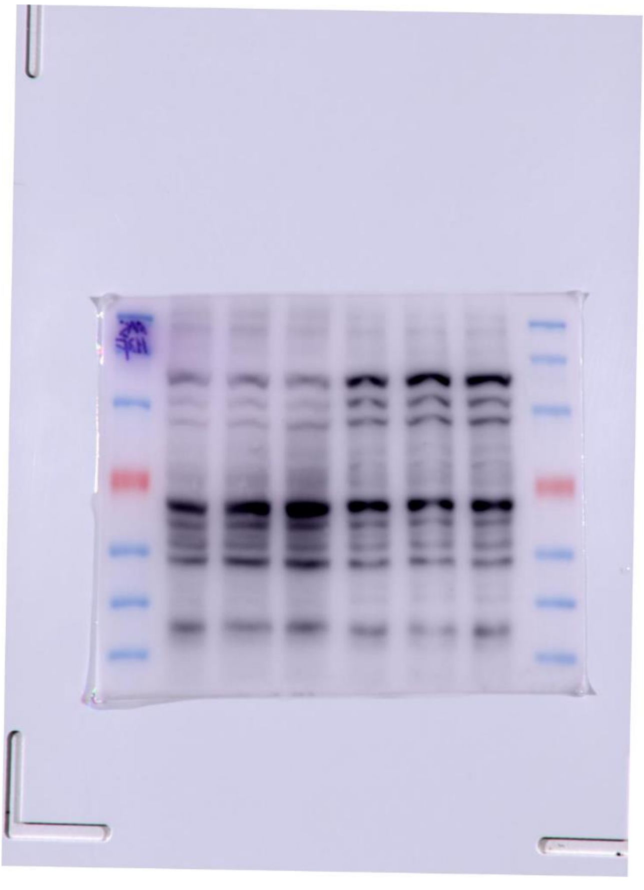

HIF-1a

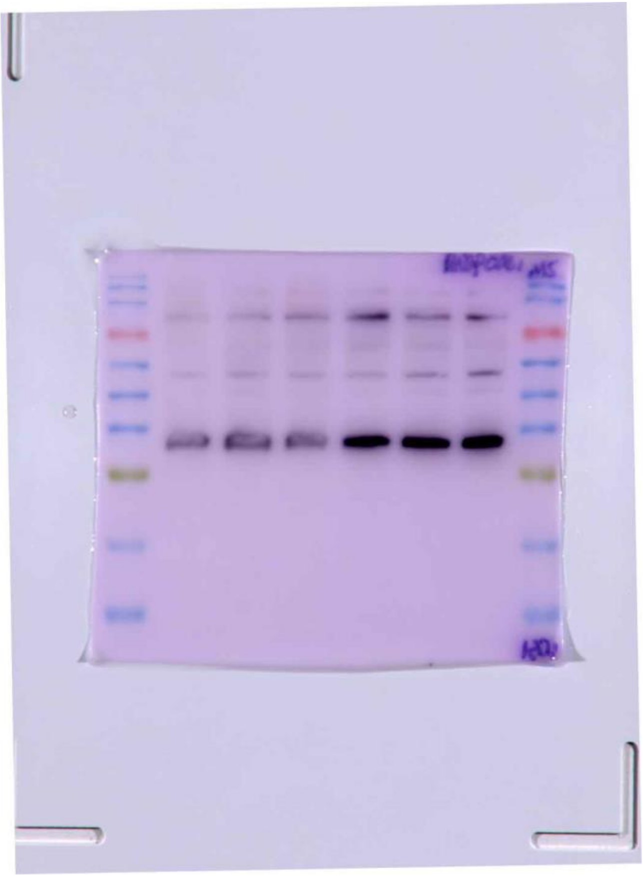

BNIP3

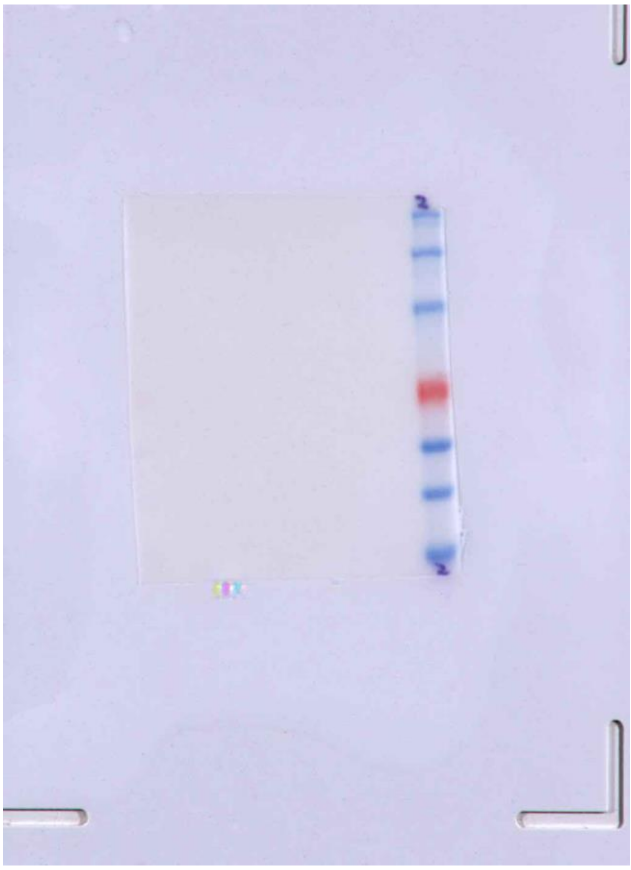

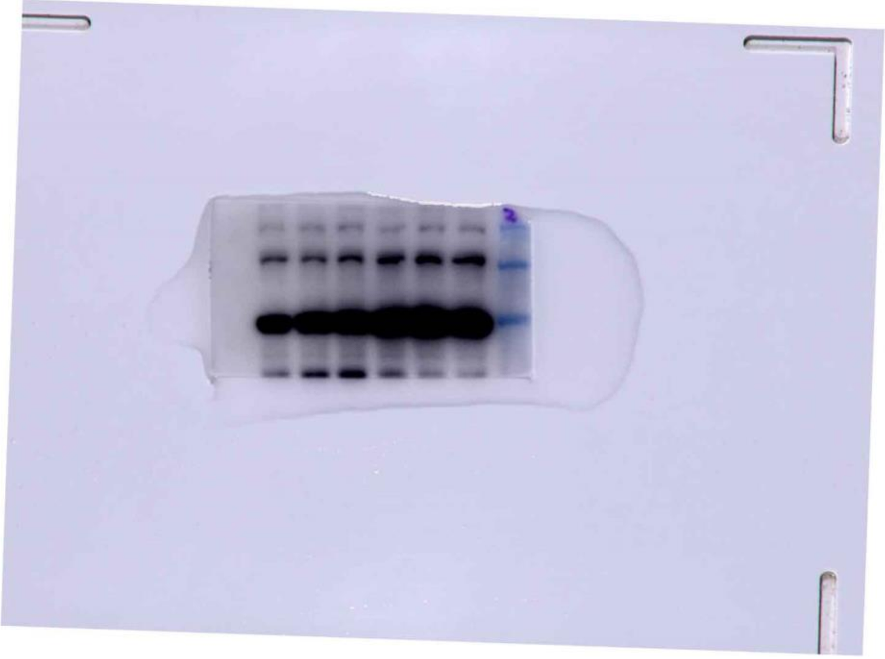

NLRP1

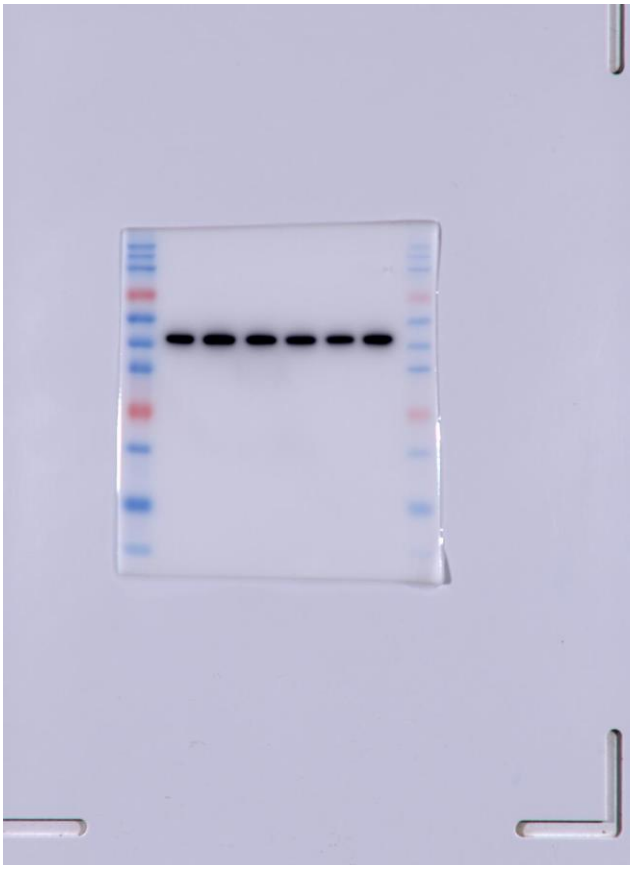

β-actin

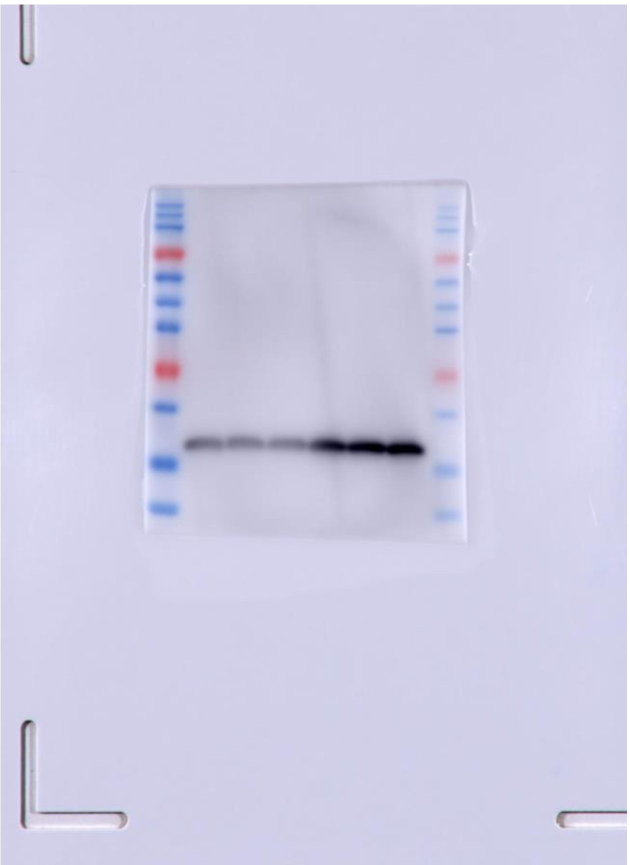

IL-1β

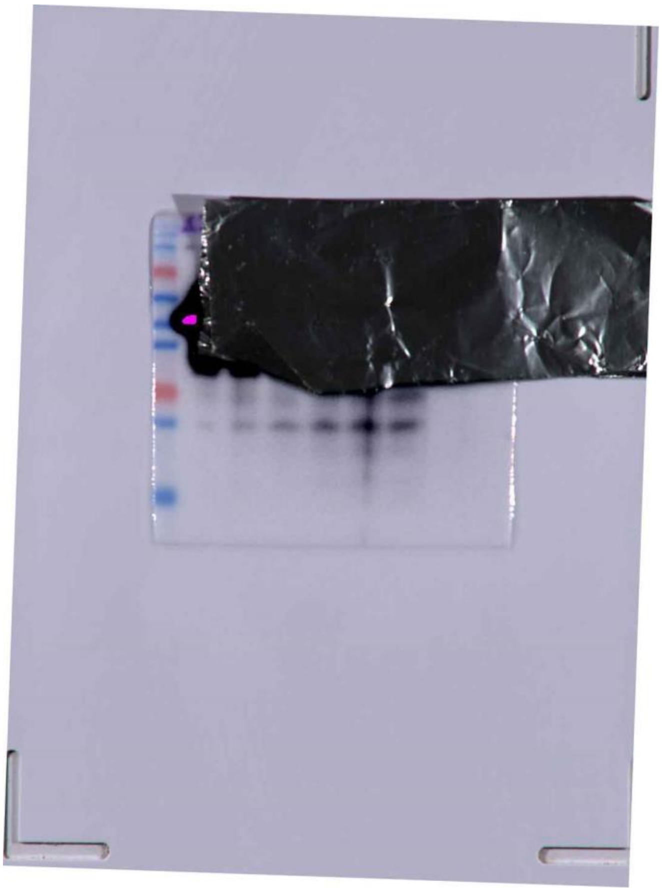

Cleaved Caspase1

Figure2

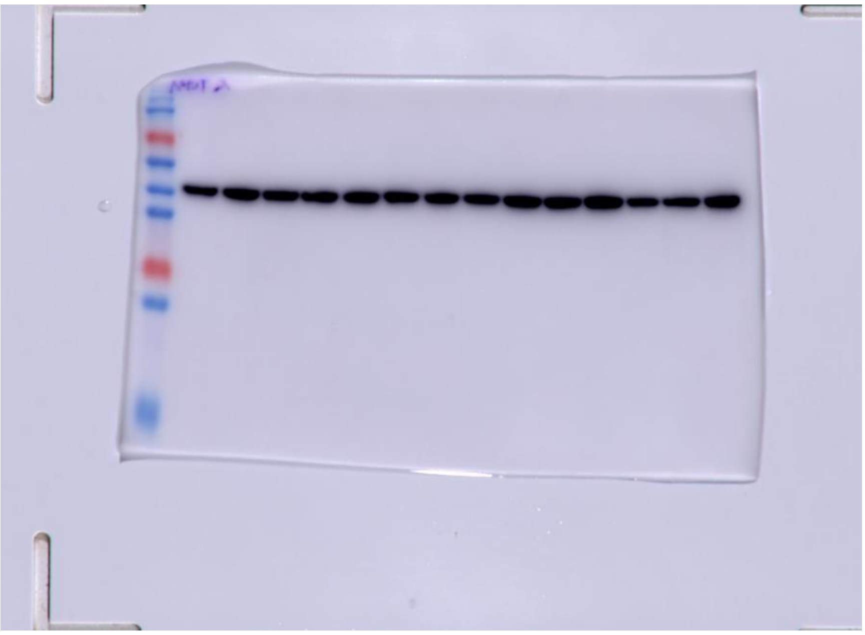

β-actin

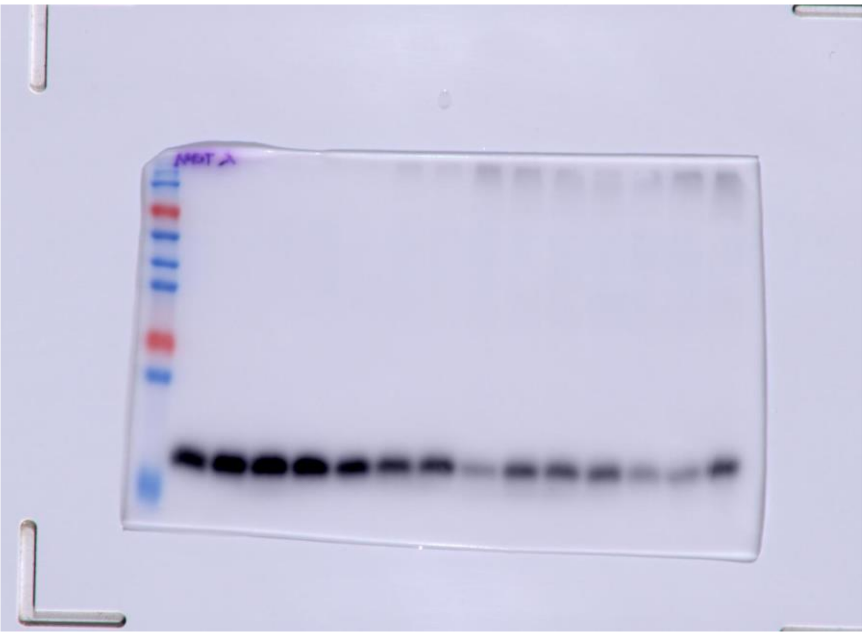

TOMM20

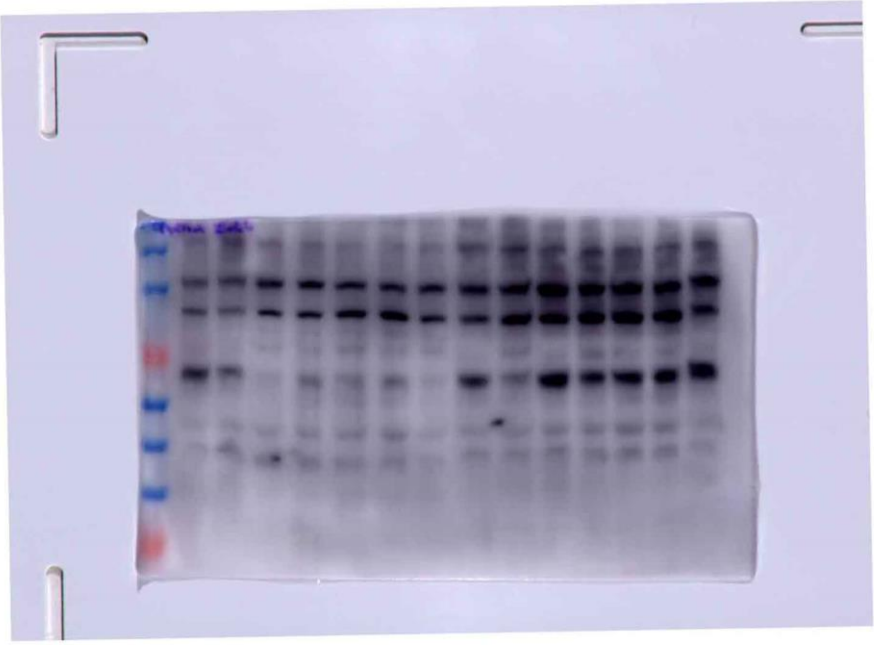

NLRP1

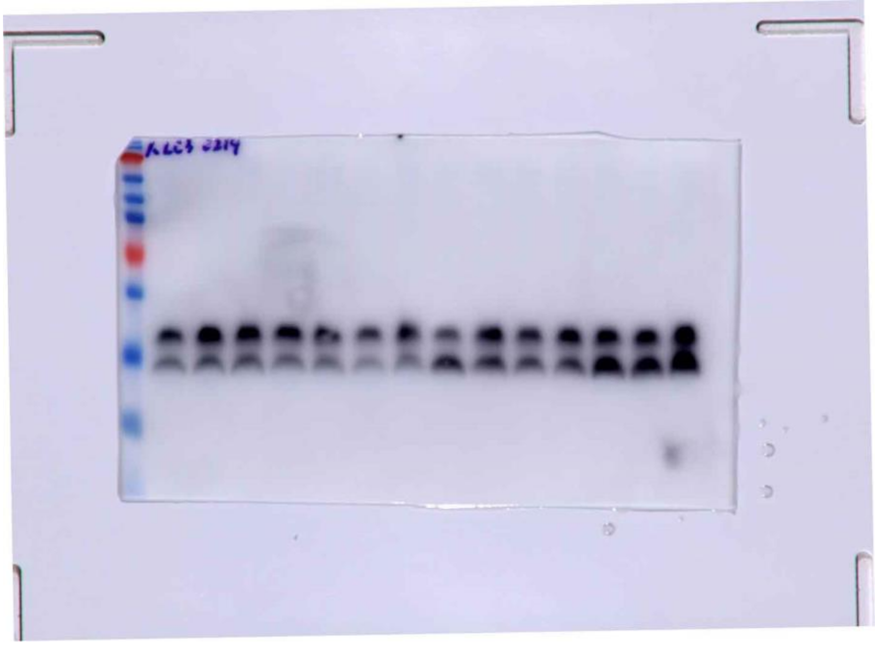

LC3

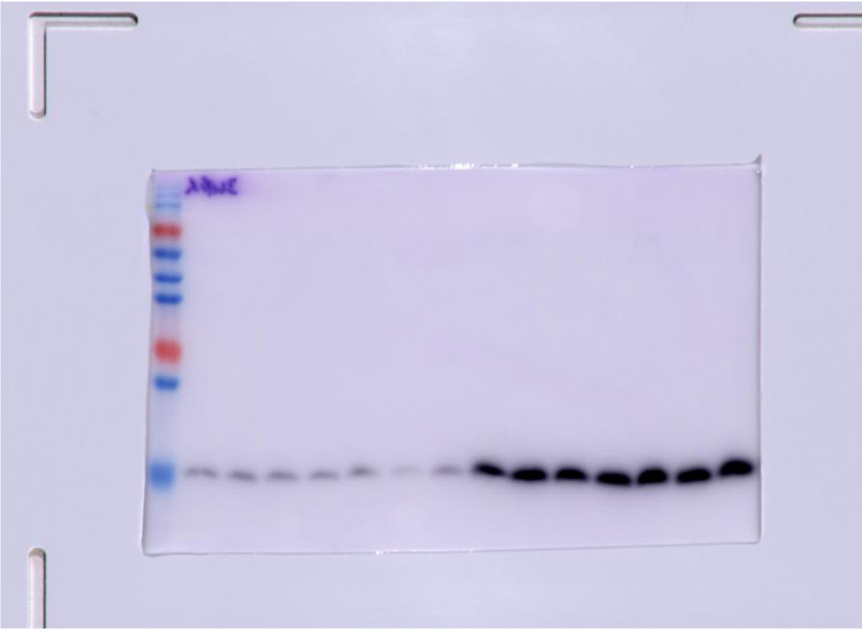

IL-1β

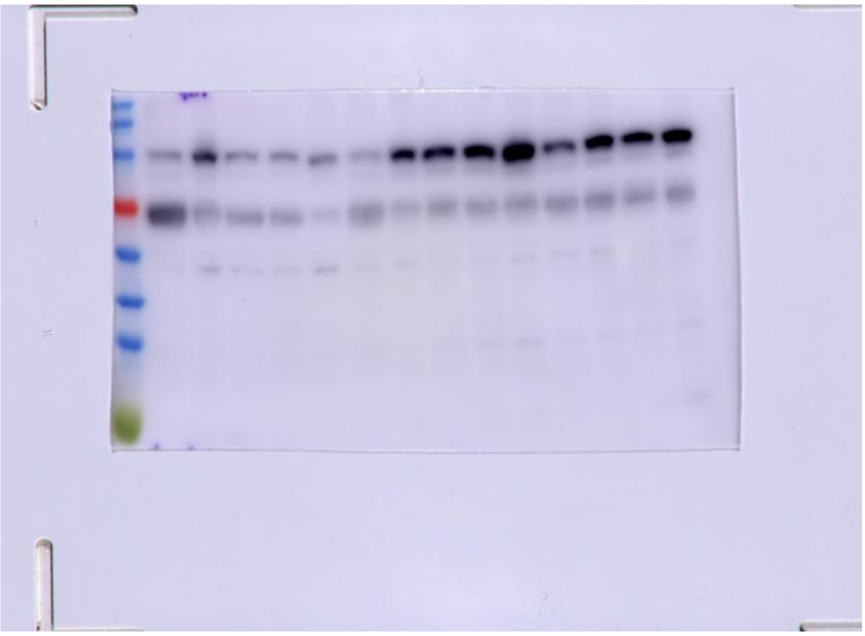

HIF-1a

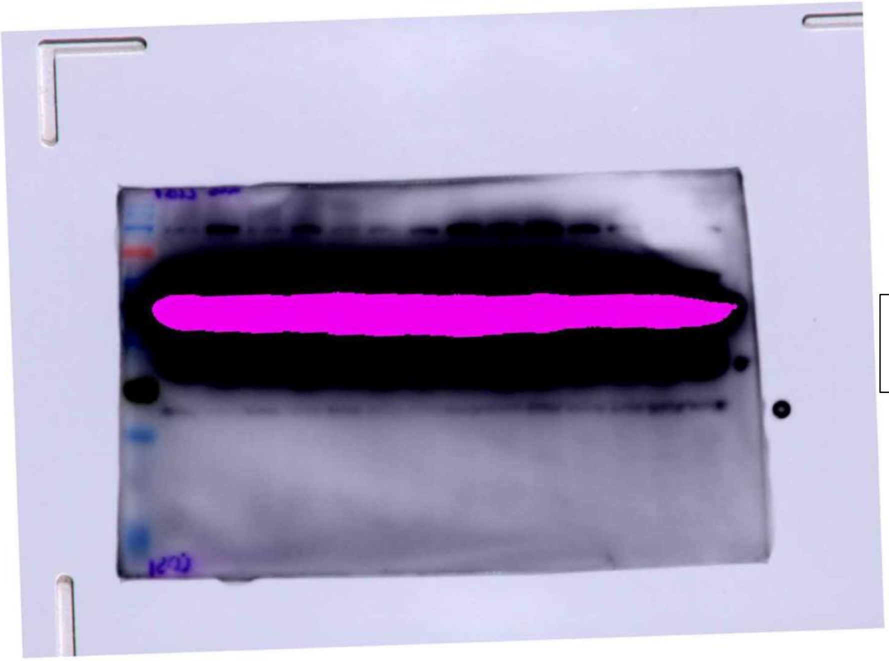

Cleaved Caspase1

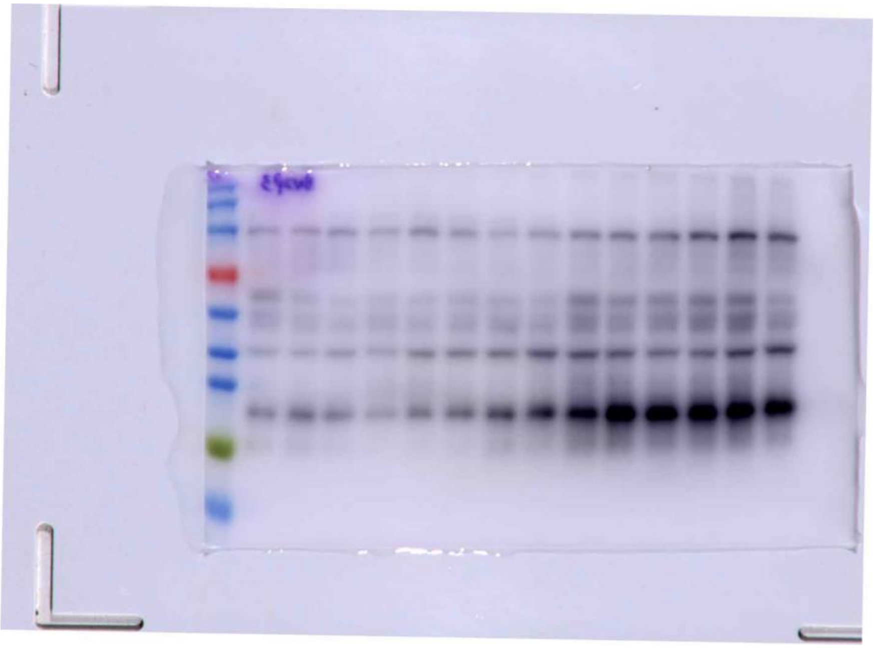

BNIP3

Figure3

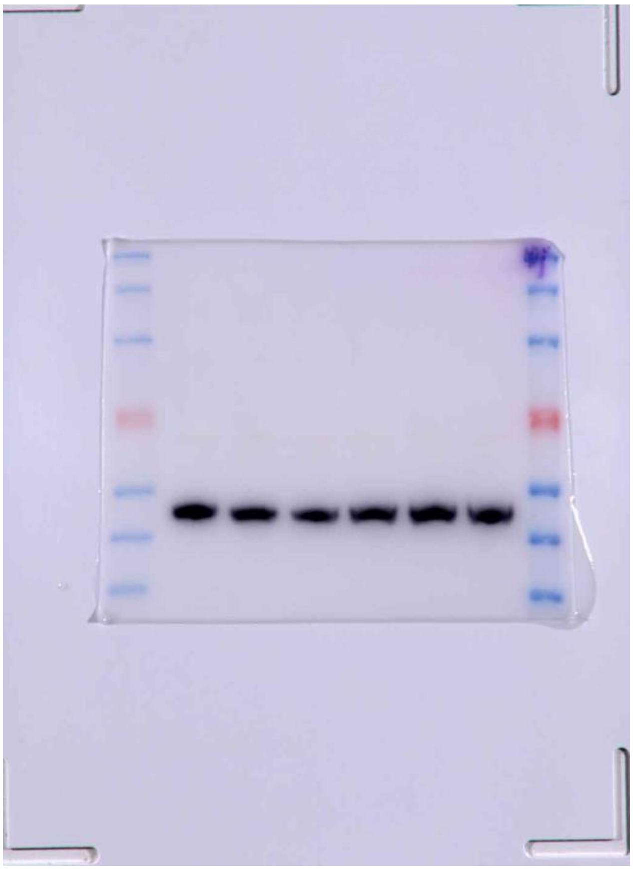

β-actin

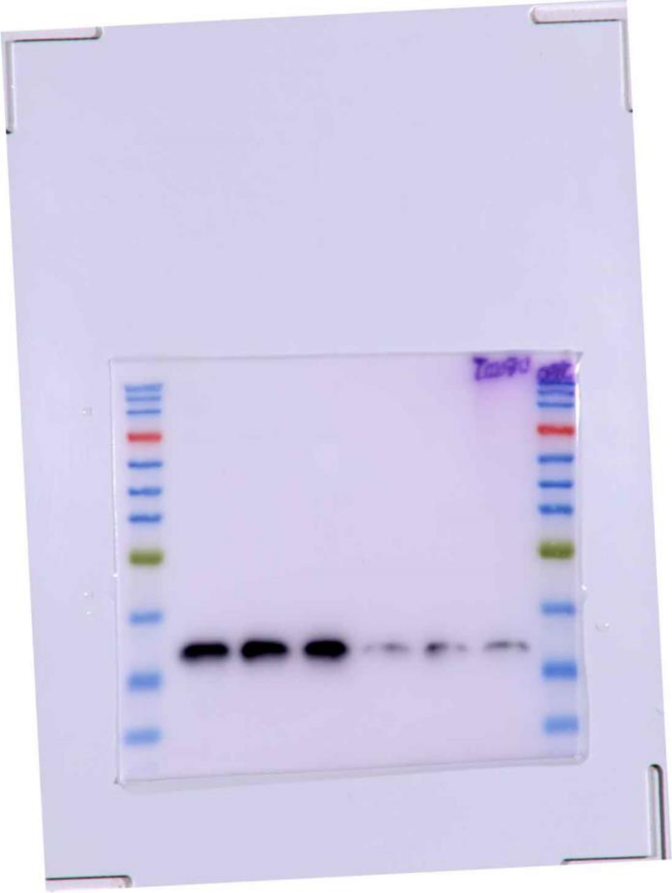

TOMM20

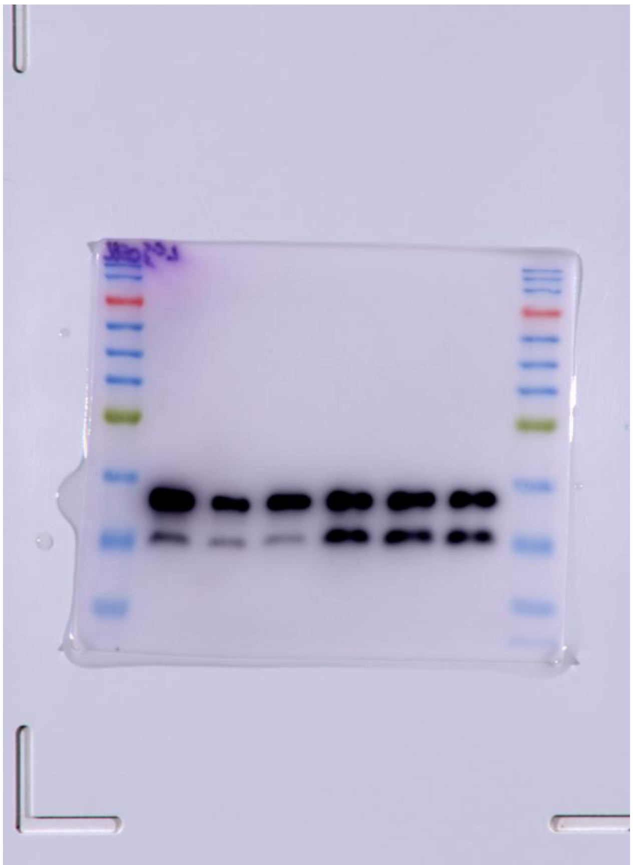

LC3

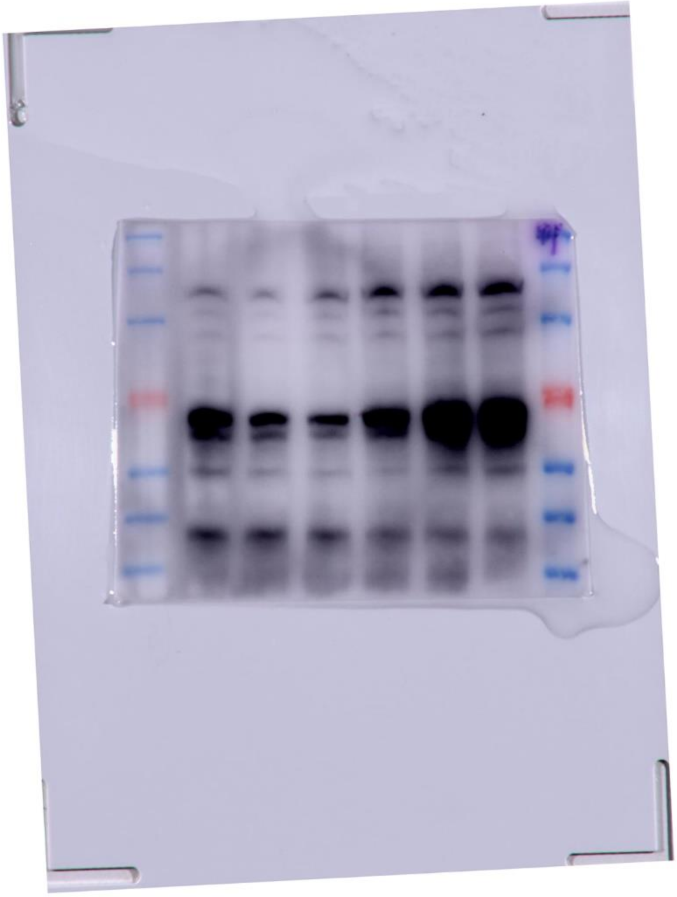

HIF-1a

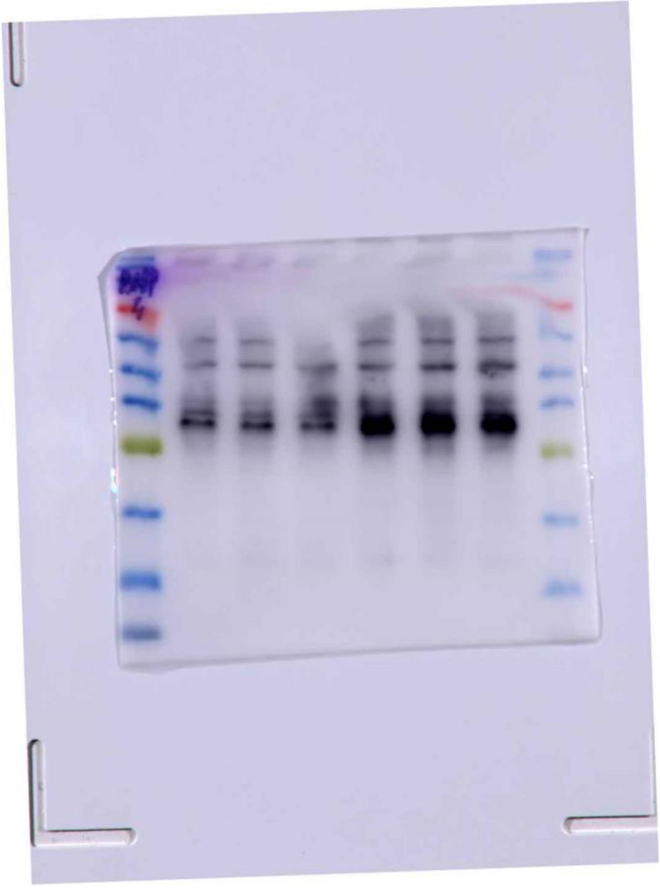

BNIP3

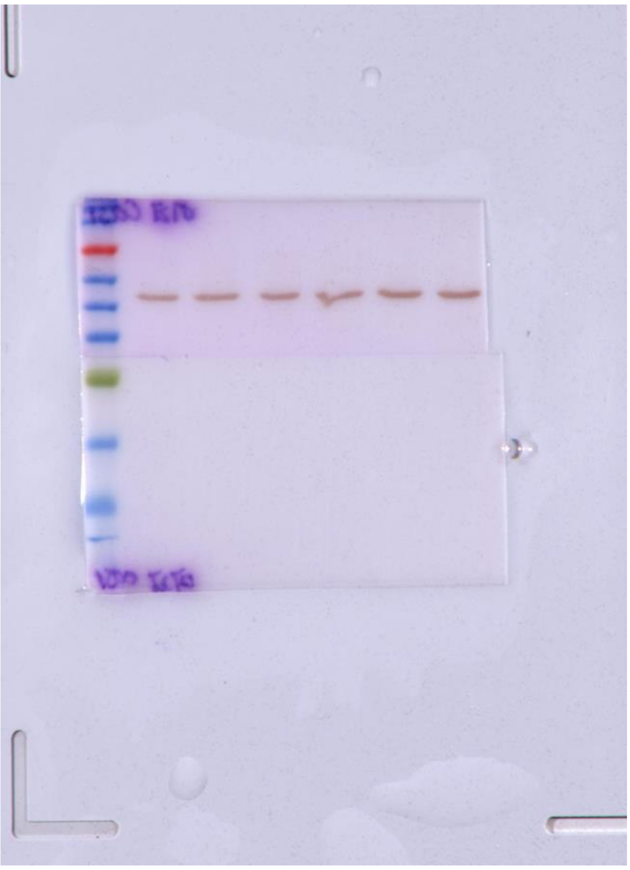

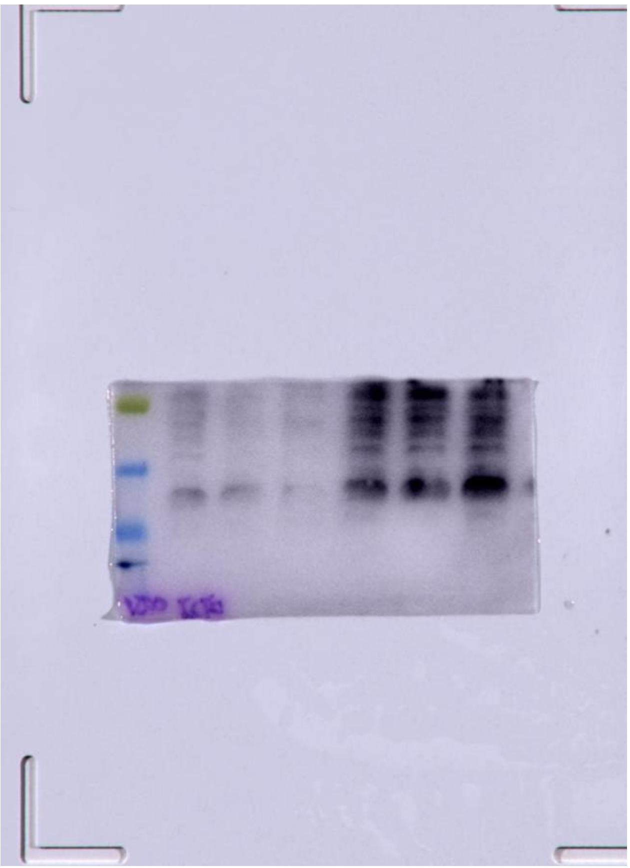

Cleaved Caspase1

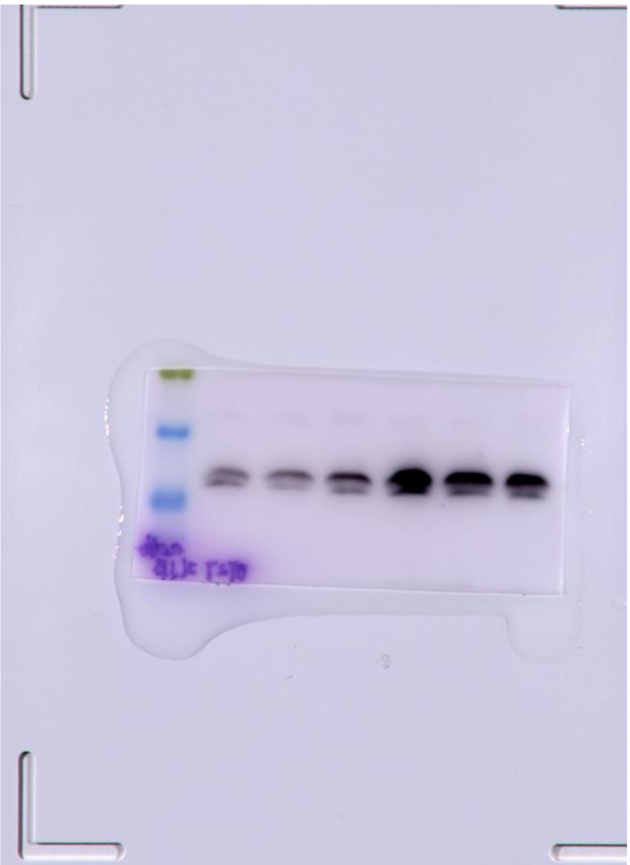

IL-1β

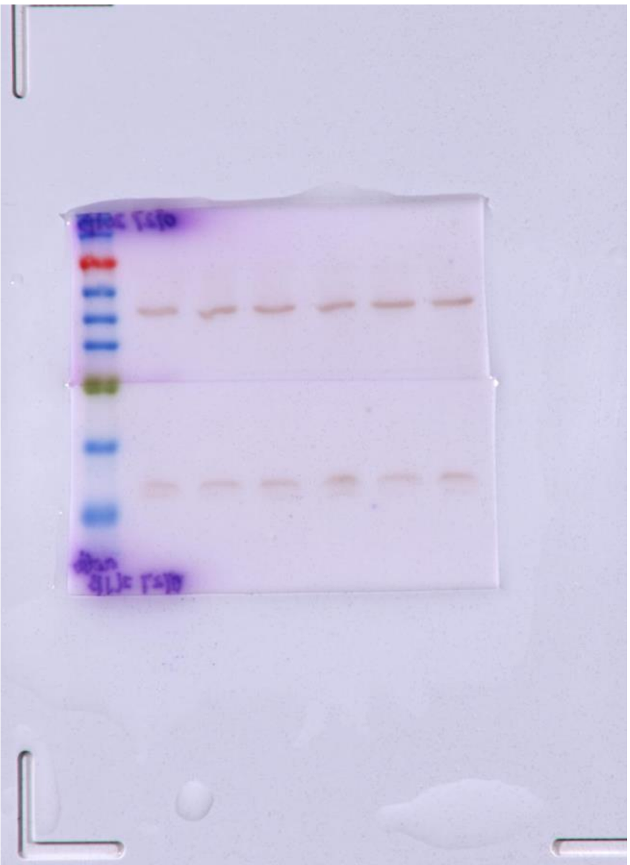

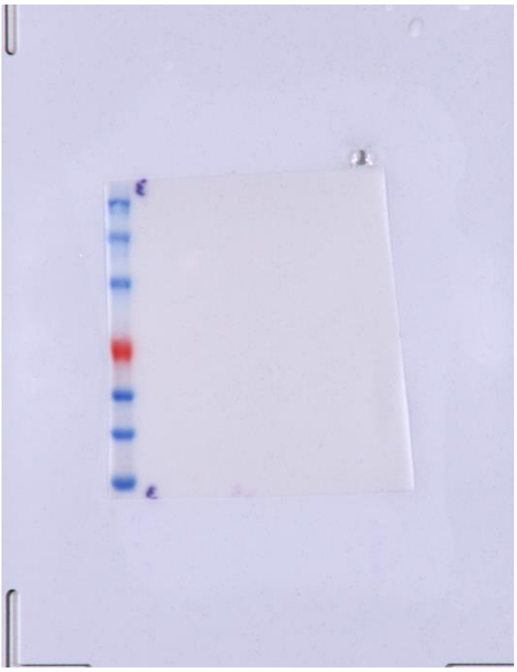

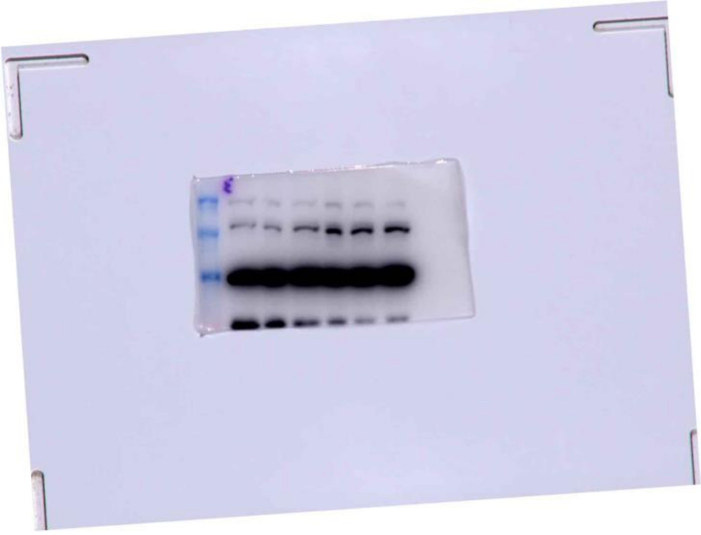

NLRP1

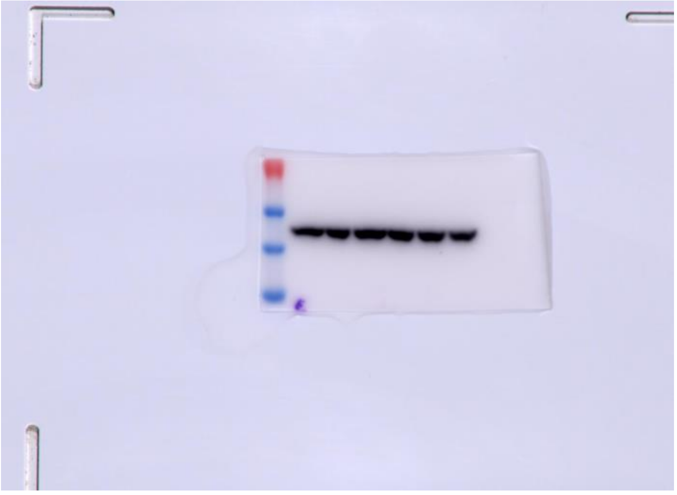

β-actin

Figure4

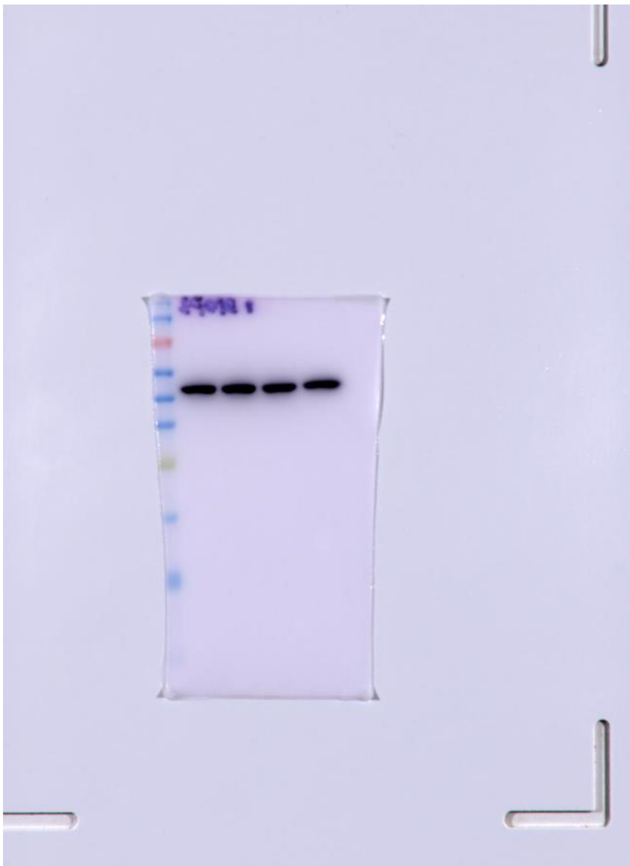

β-actin

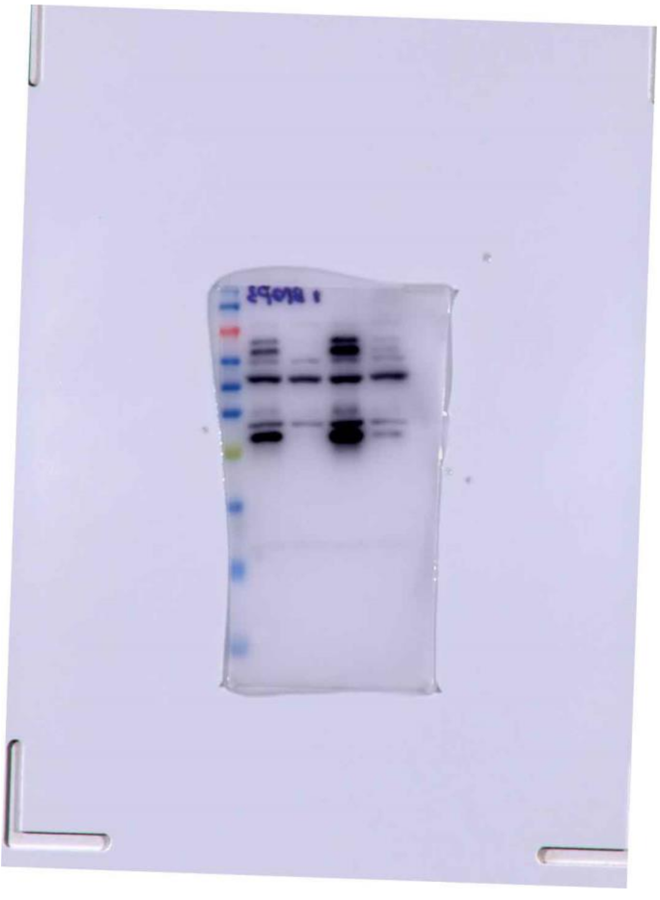

BNIP3

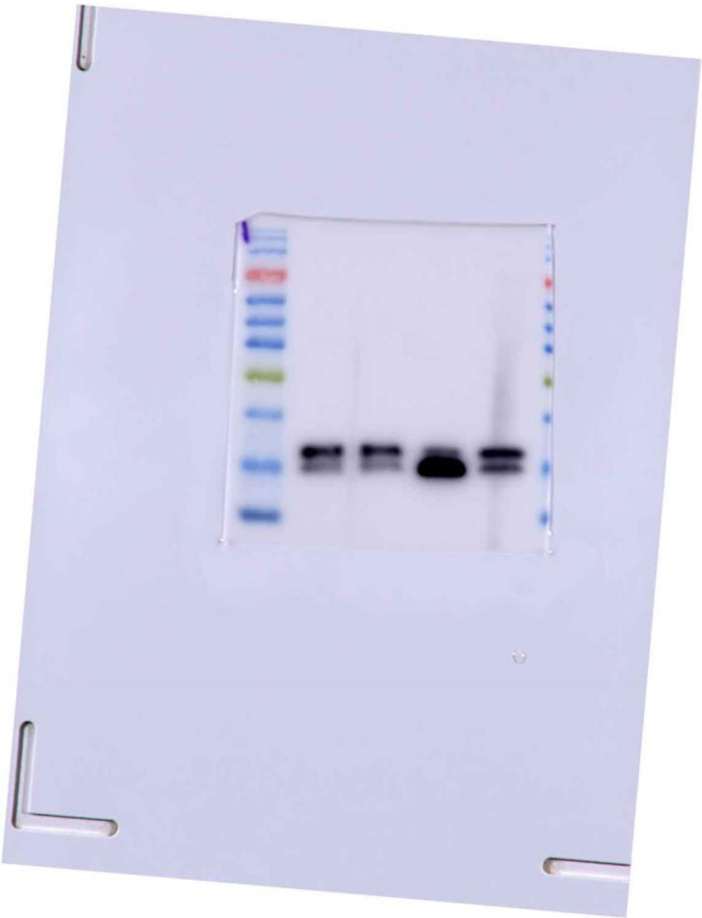

LC3

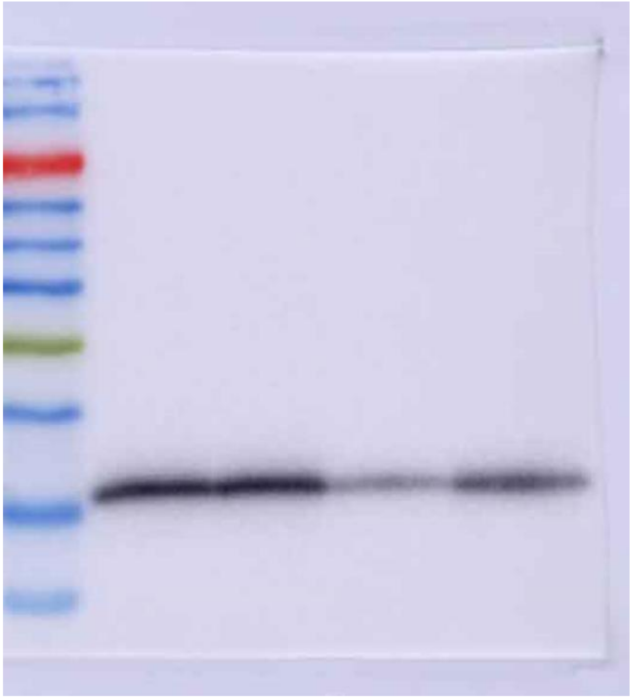

Tomm20

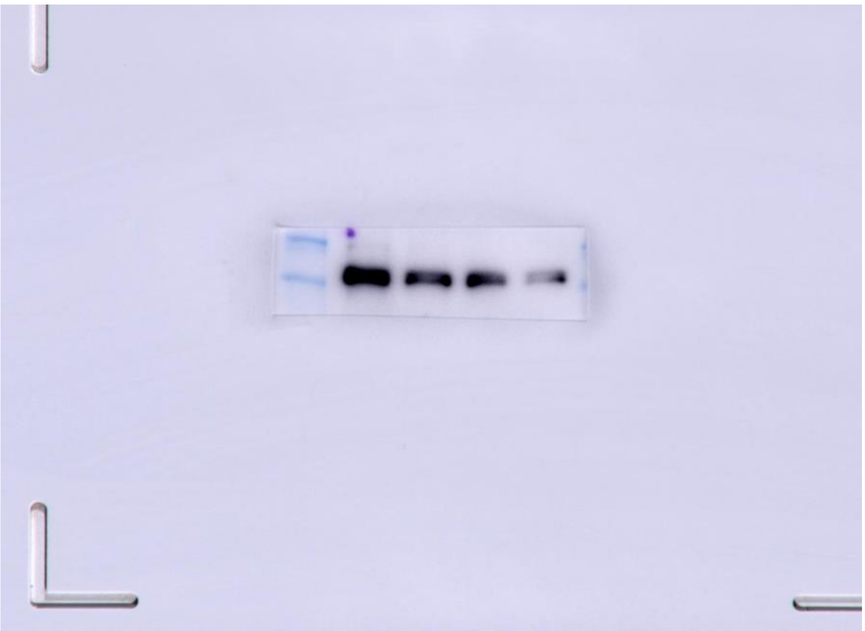

NLRP1

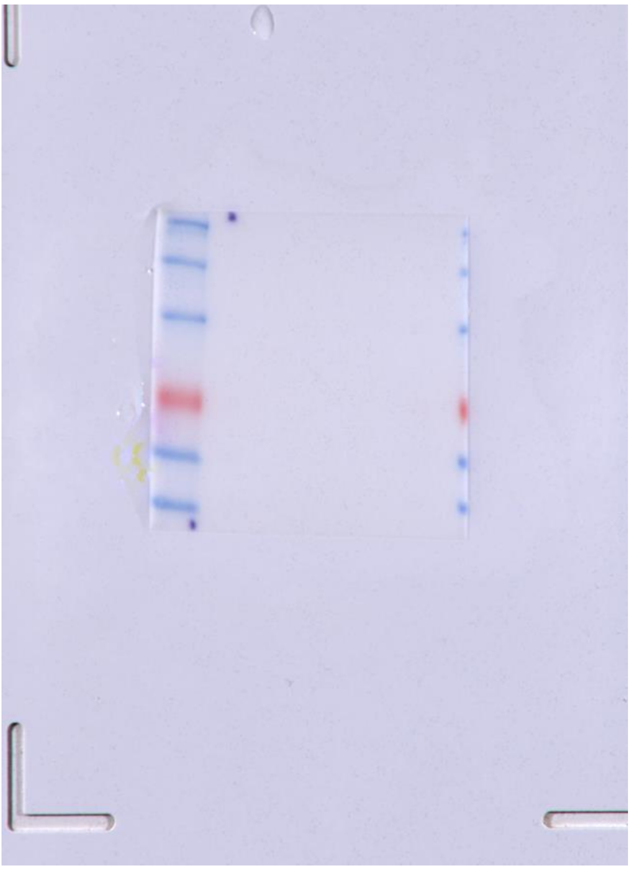

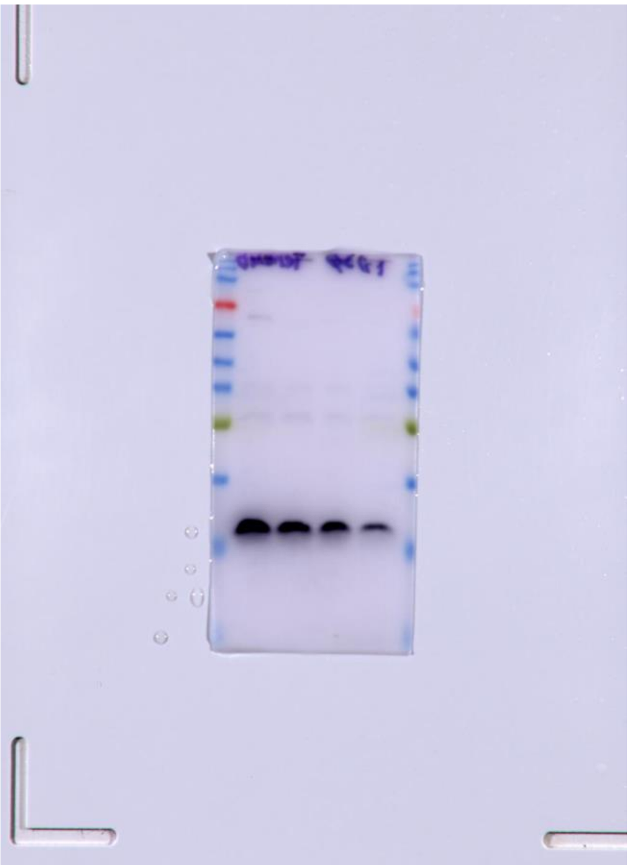

IL-1β

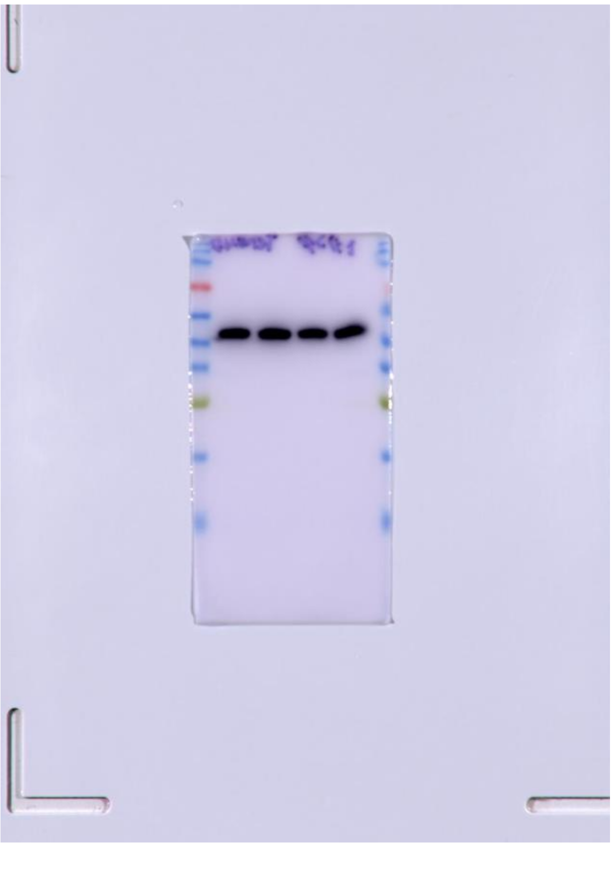

β-actin

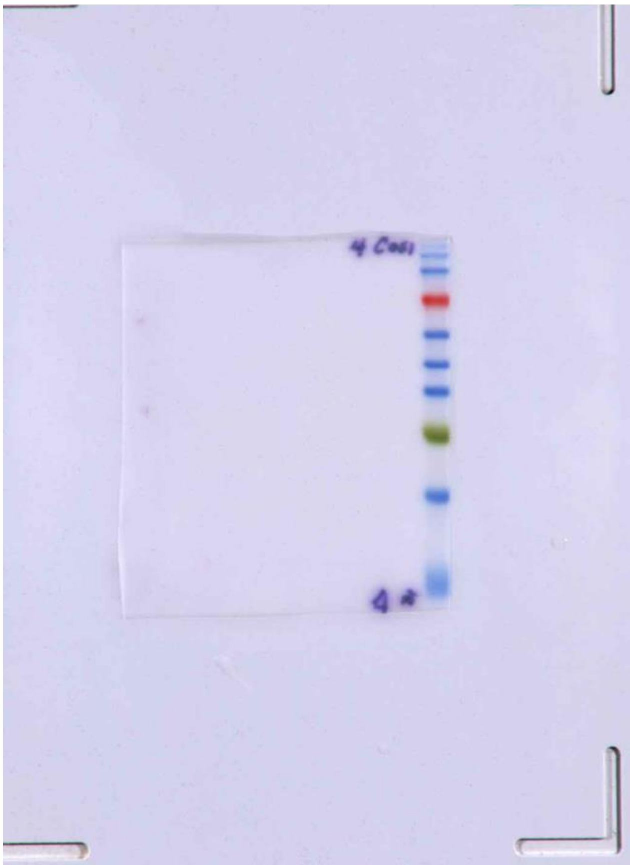

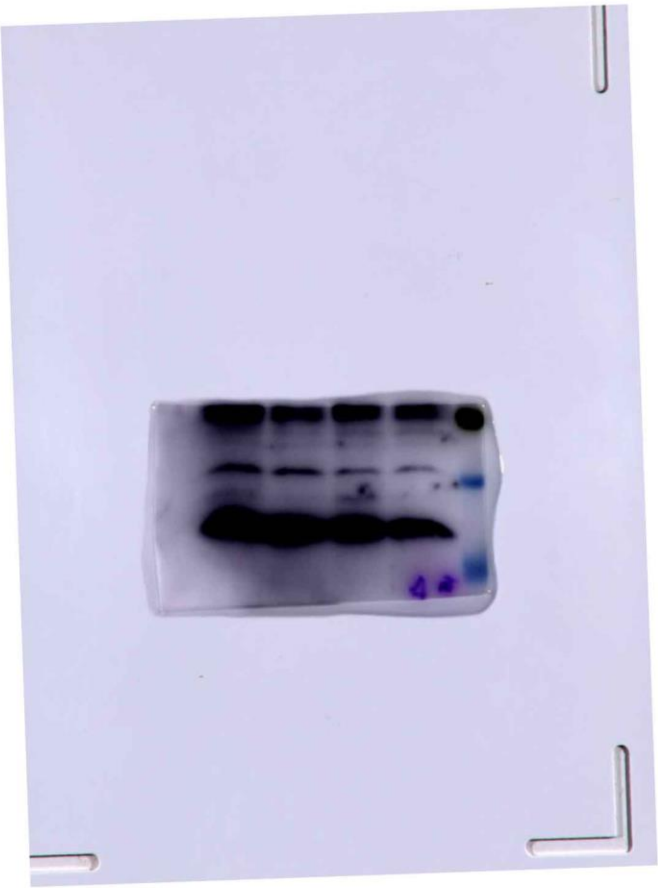

Cleaved Caspase1

Figure5

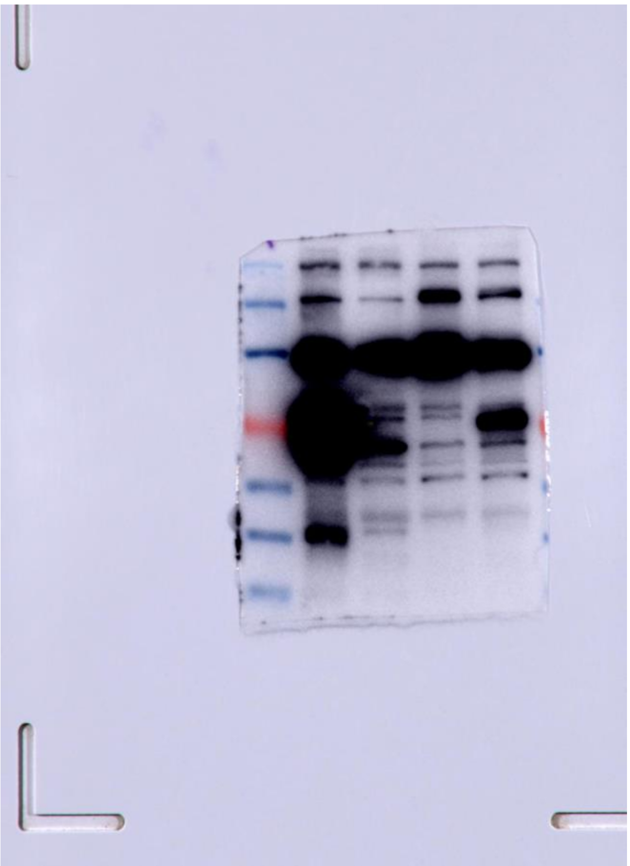

NLRP1

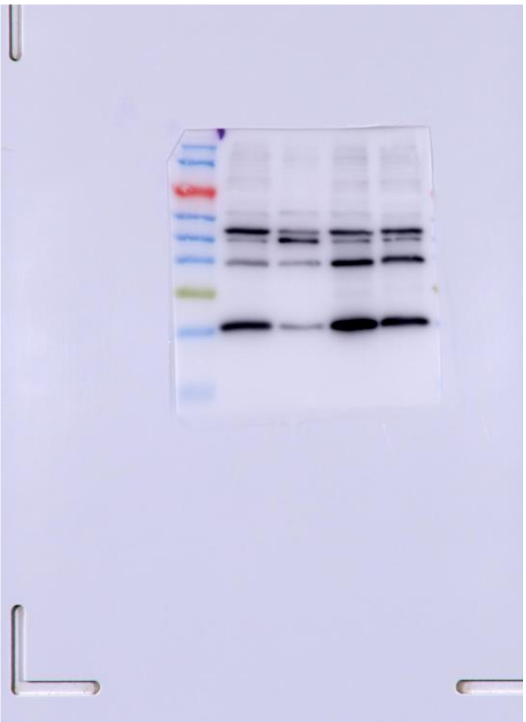

Cleaved Caspase1

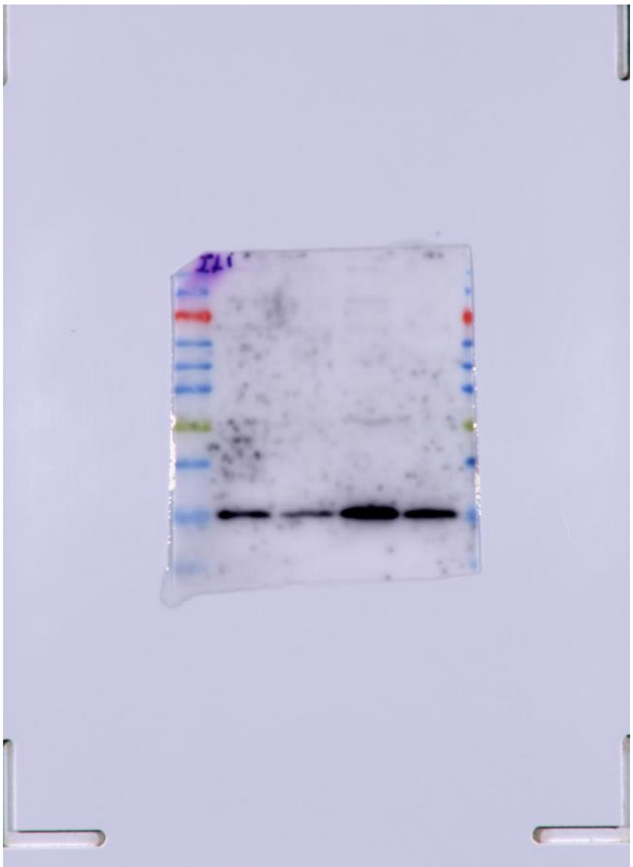

IL-1β

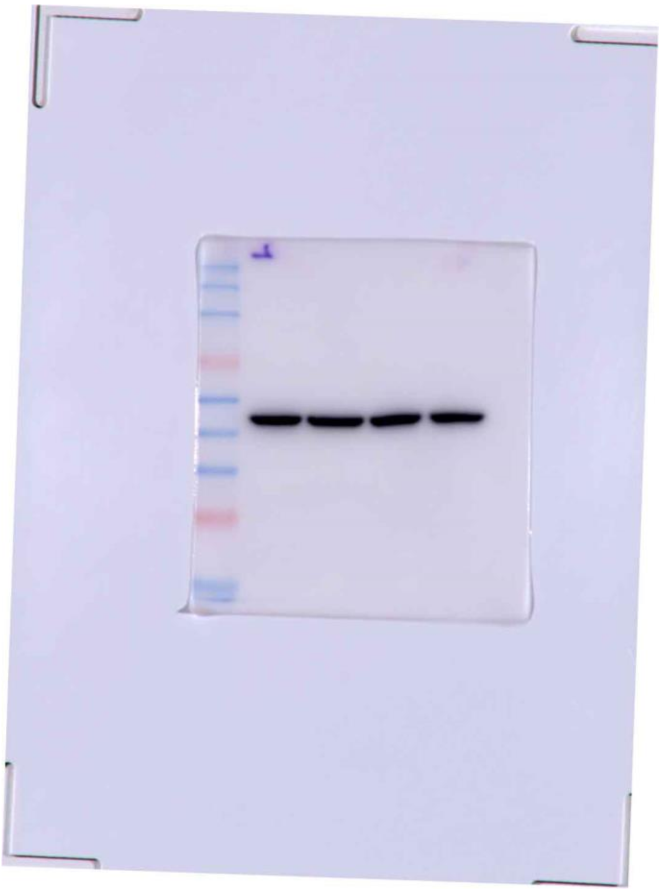

β-actin

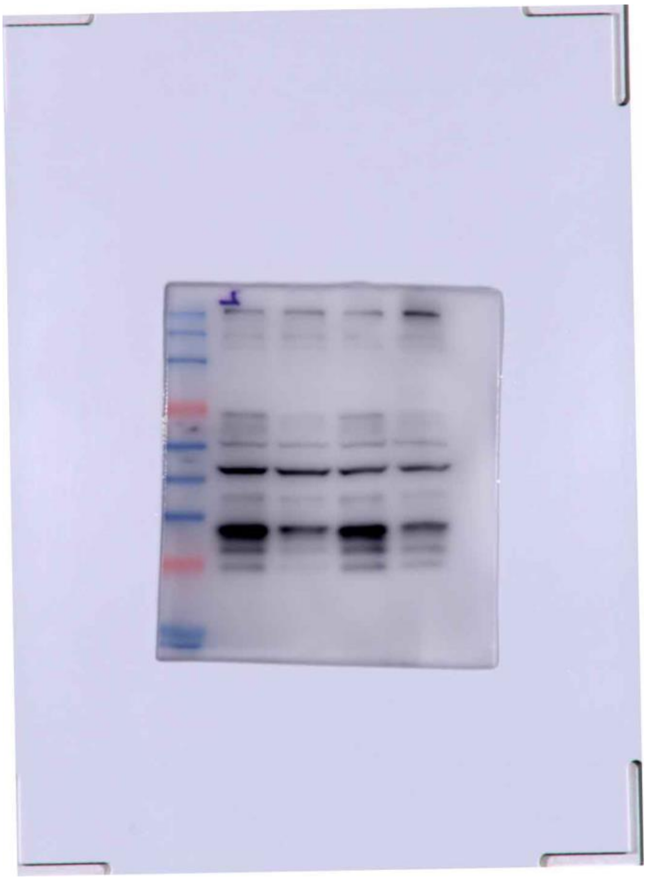

BNIP3

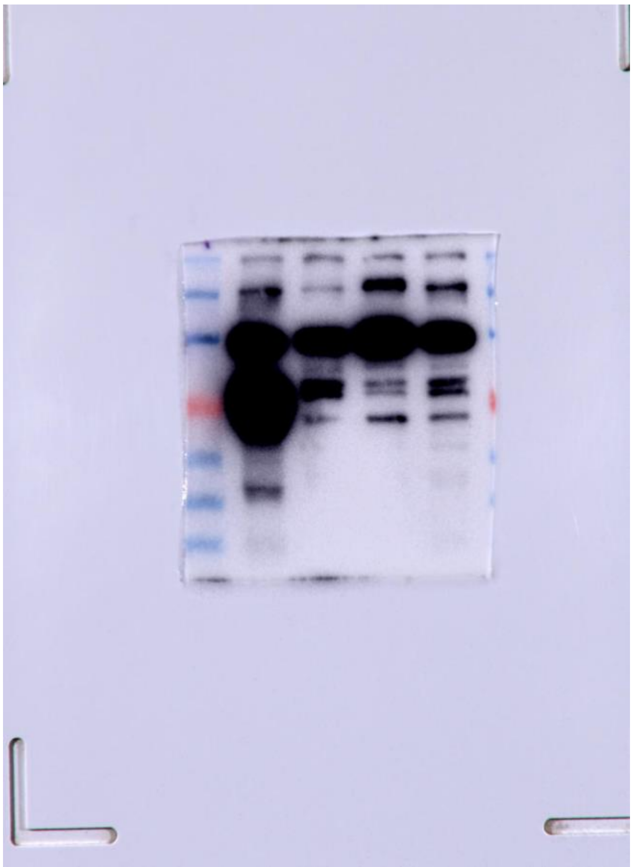

NLRP1

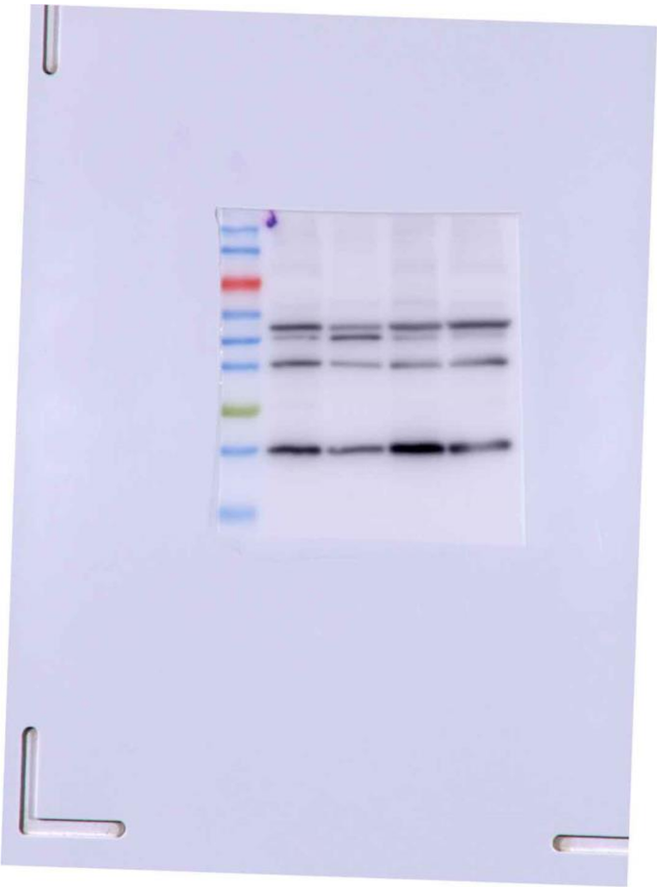

Cleaved Caspase1

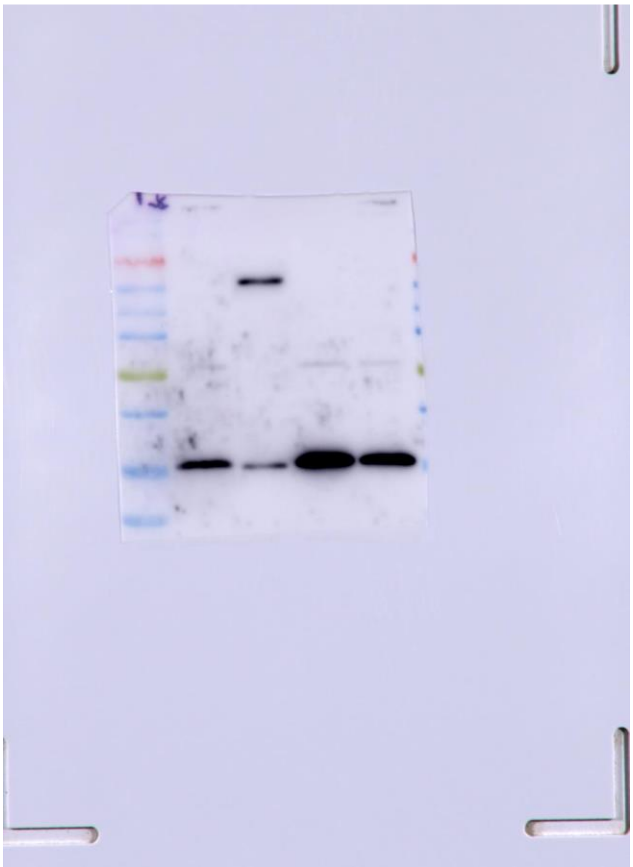

IL-1β

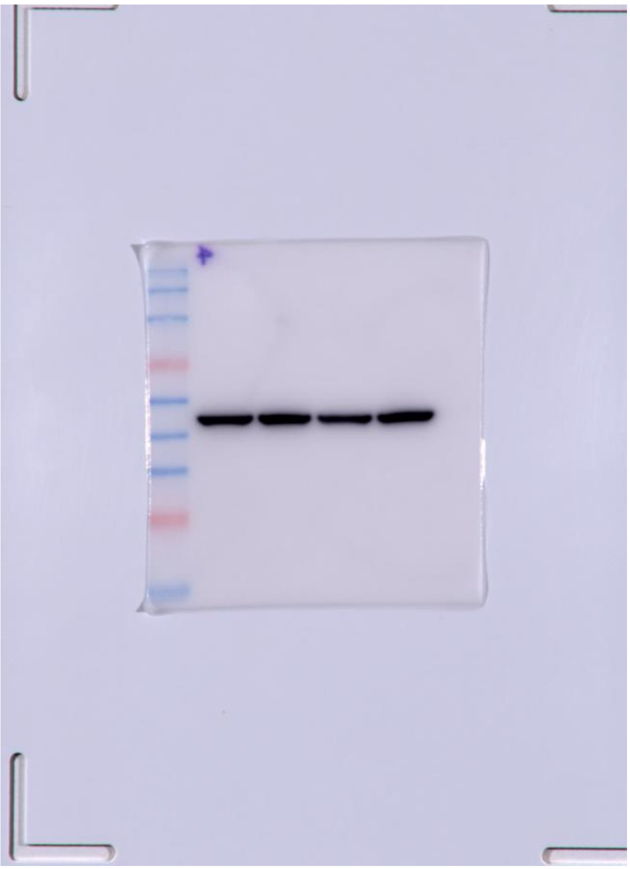

β-actin

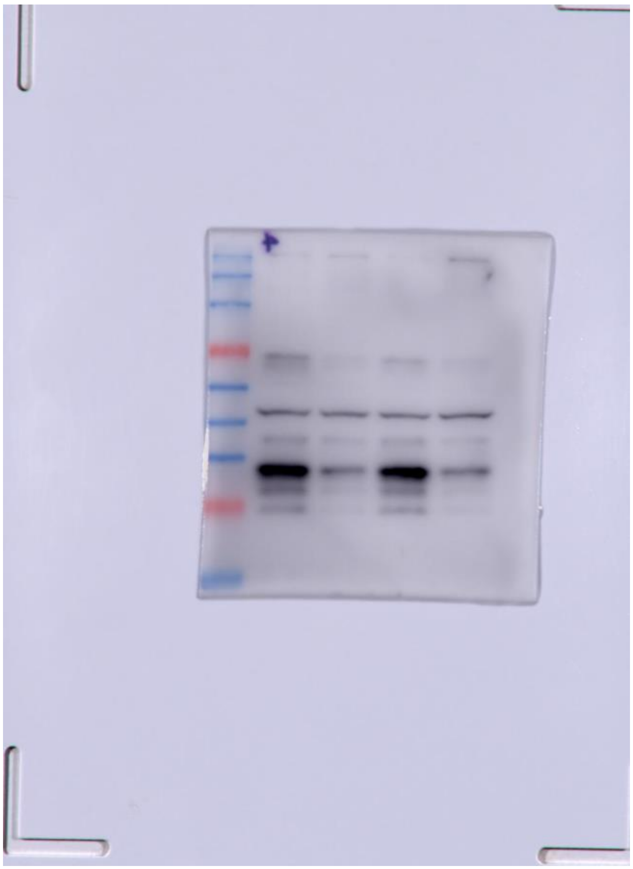

BNIP3

Figure6

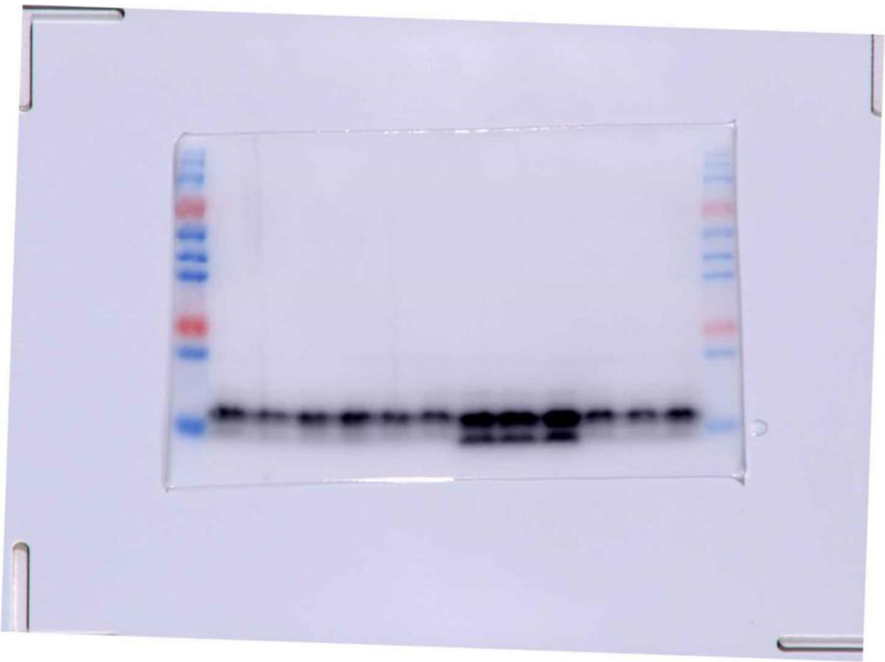

LC3

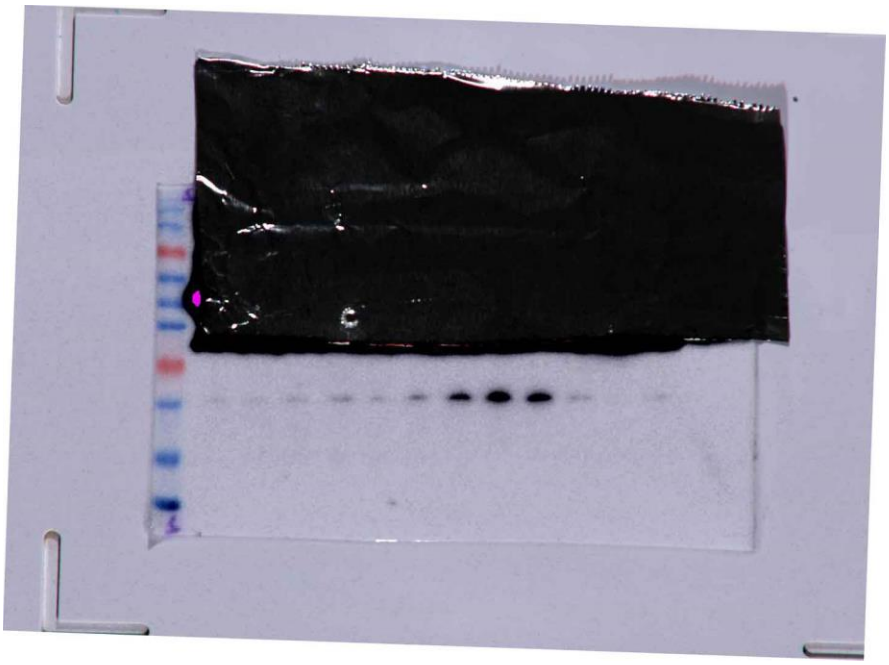

Cleaved Caspase1

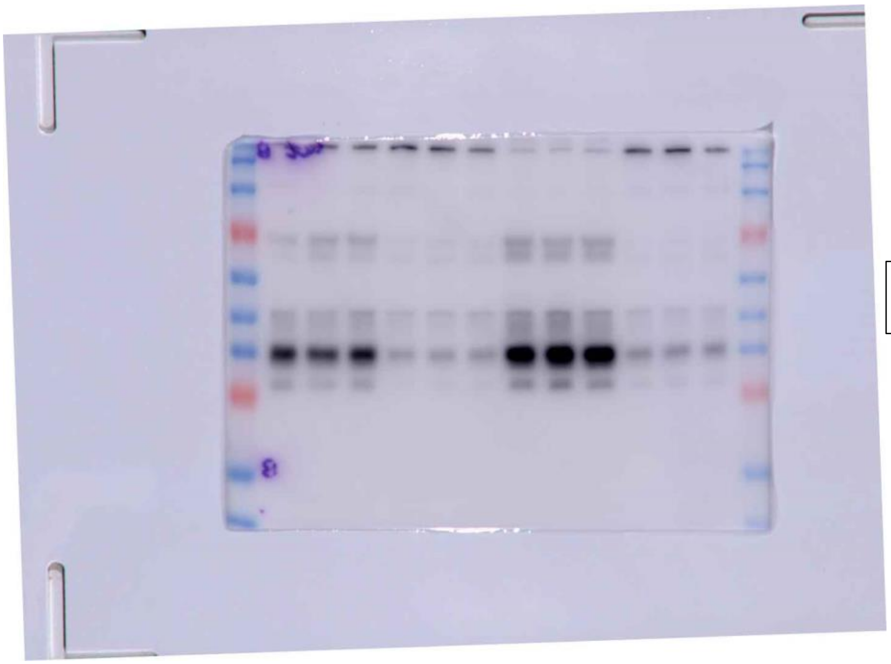

BNIP3

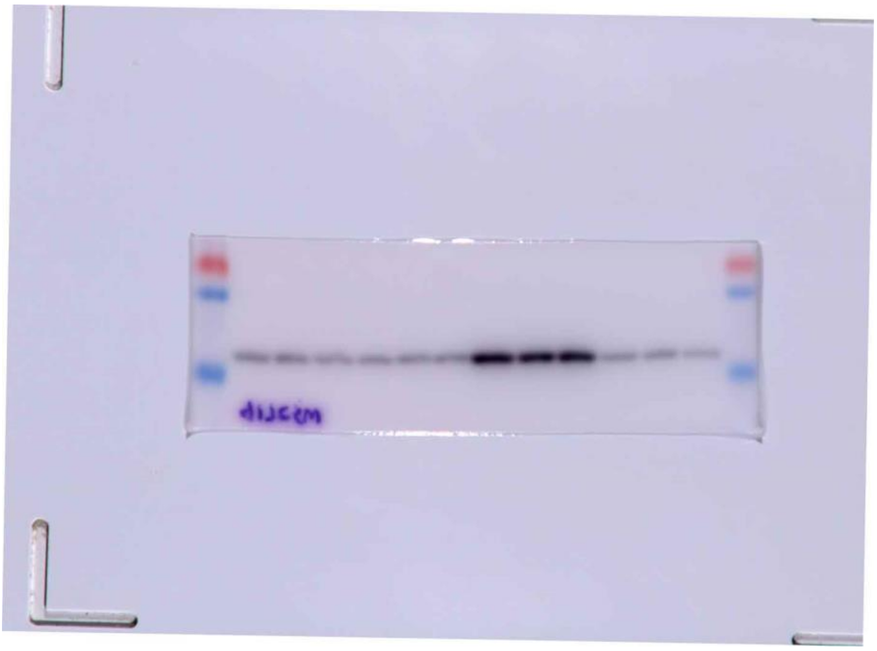

IL-1β

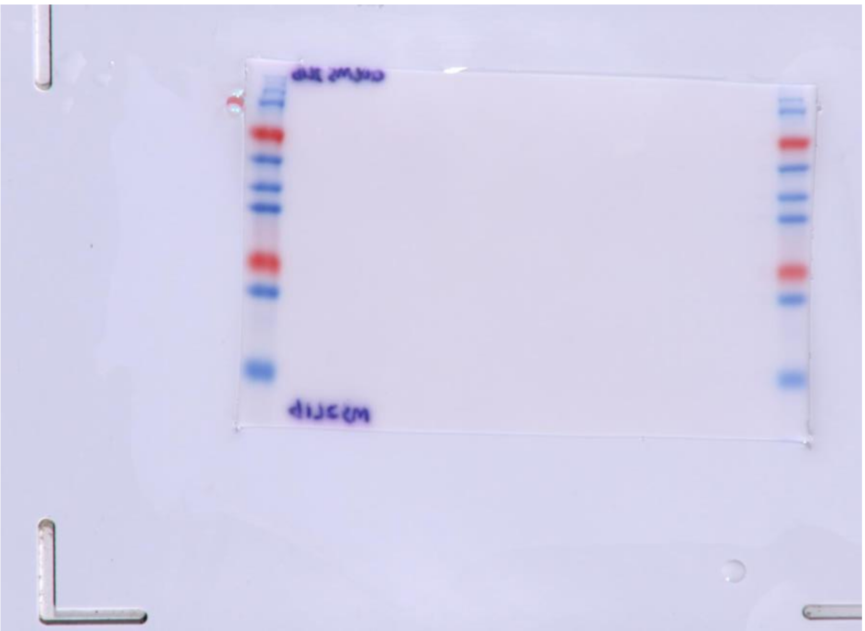

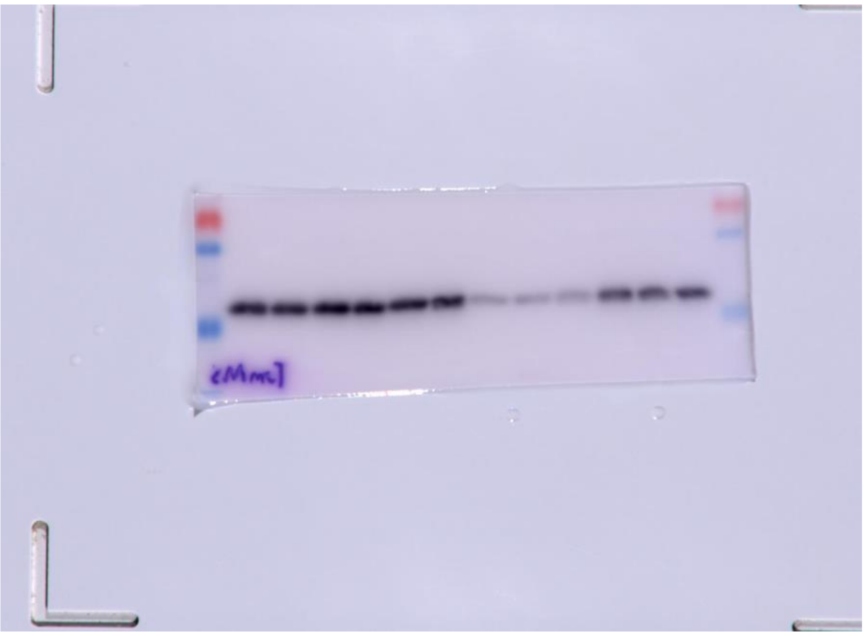

TOMM20

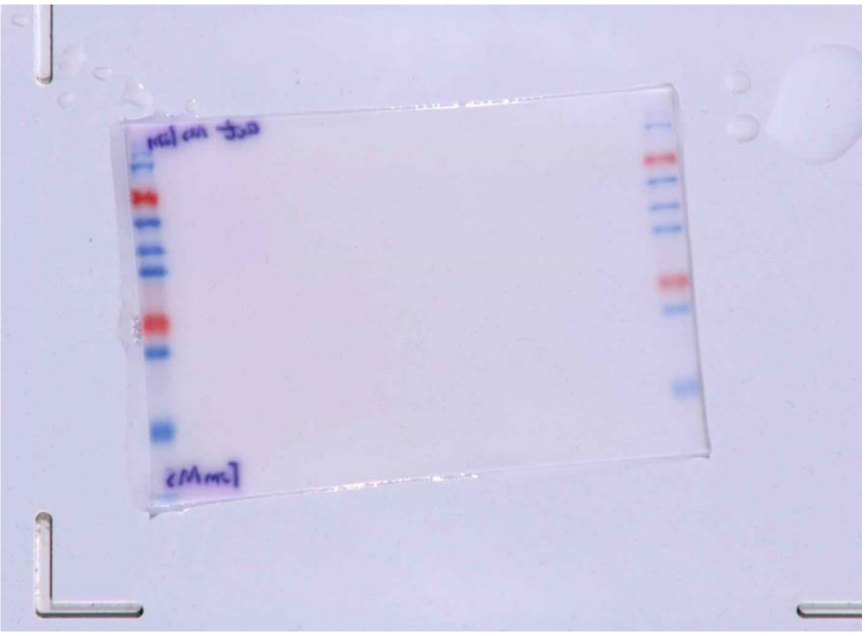

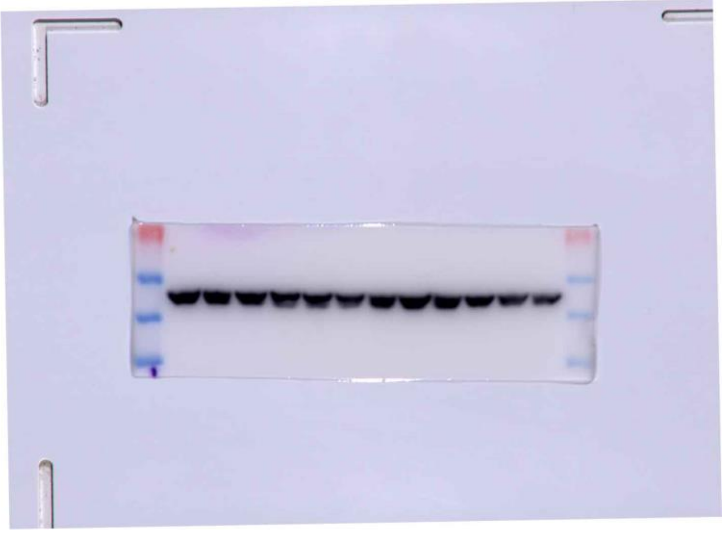

β-actin

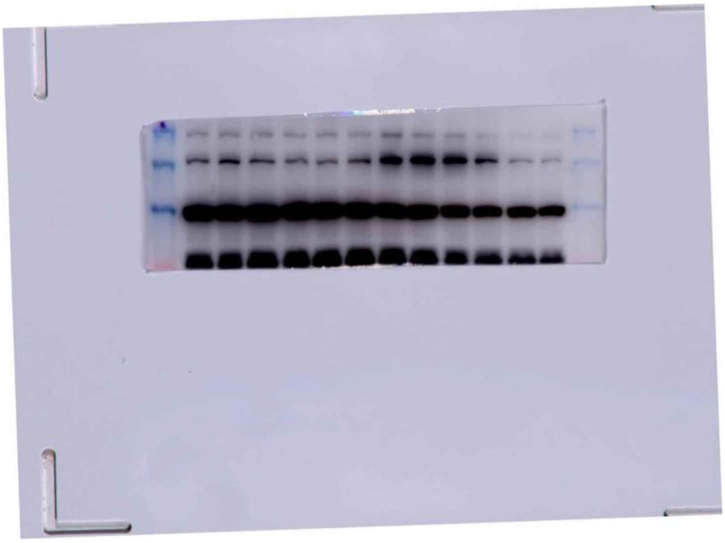

NLRP1

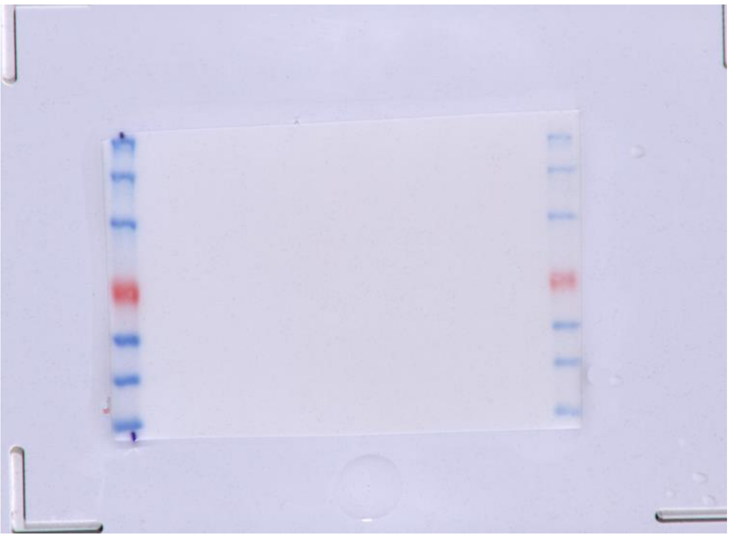

Supplementary Figure3

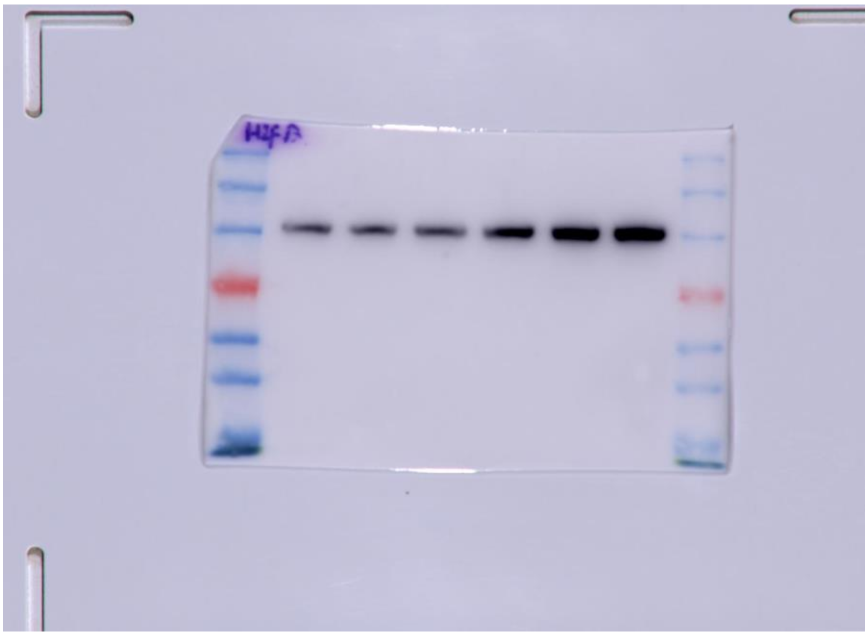

HIF-1a

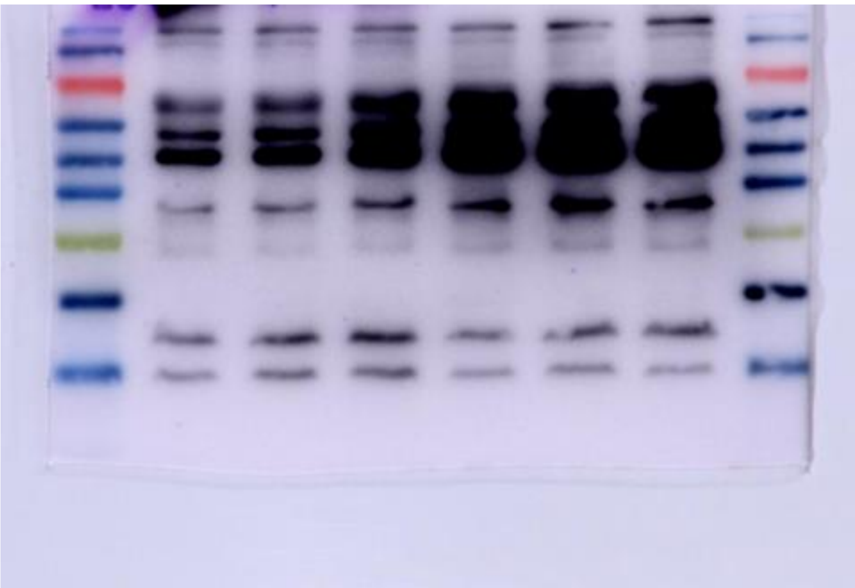

BNIP3

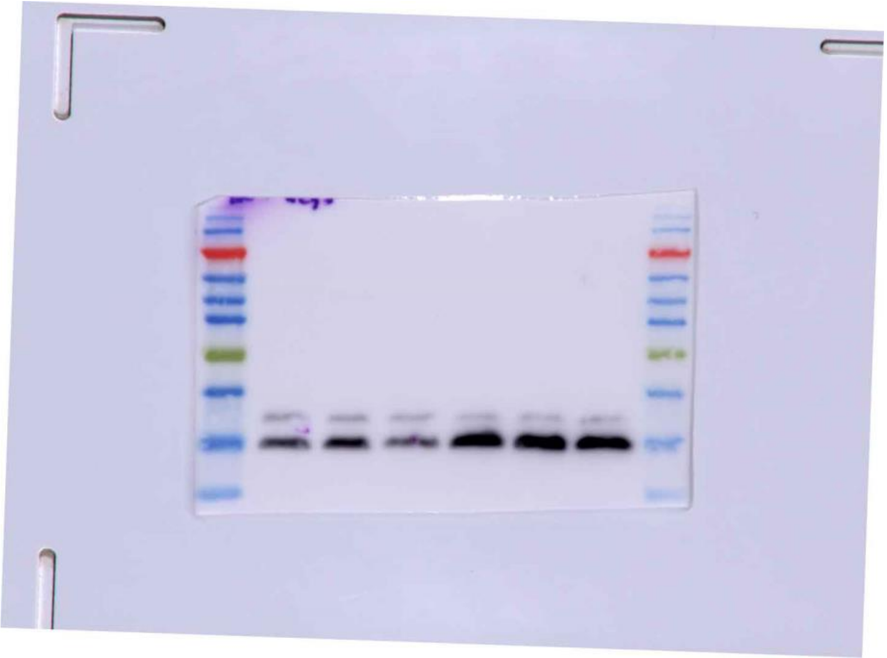

LC3

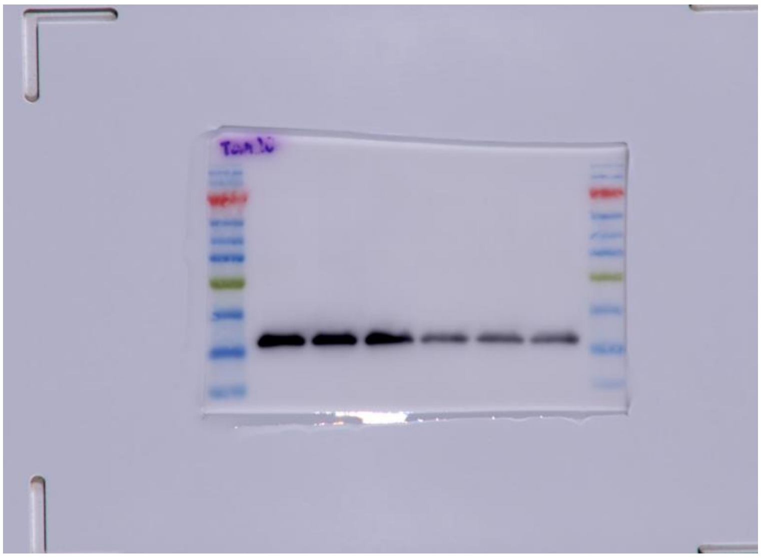

TOMM20

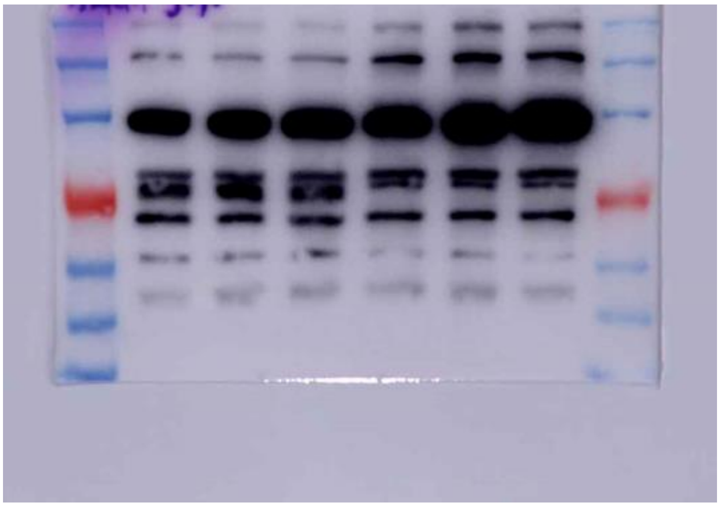

NLRP1

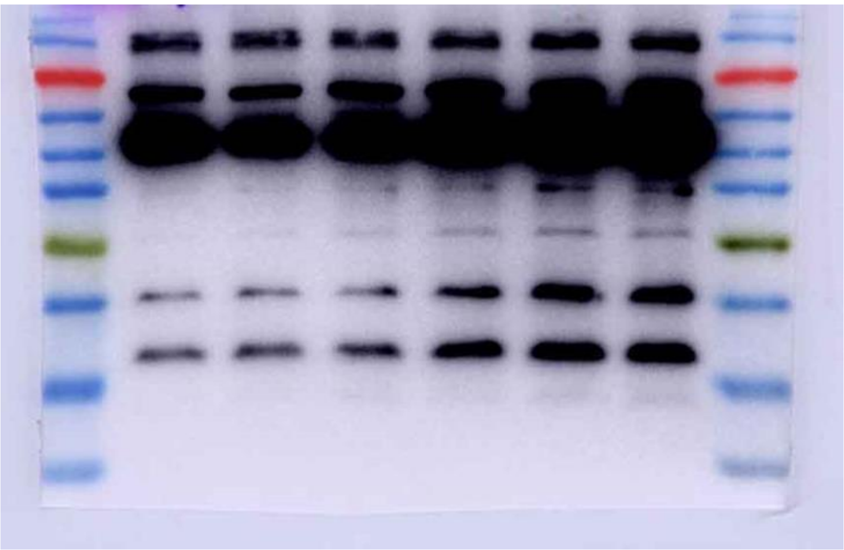

Cleaved Caspase1

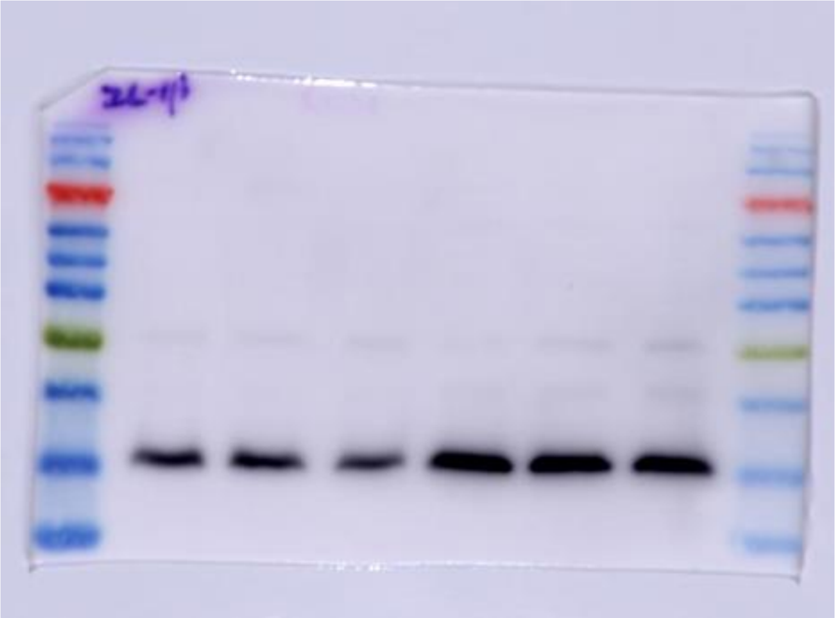

IL-1b

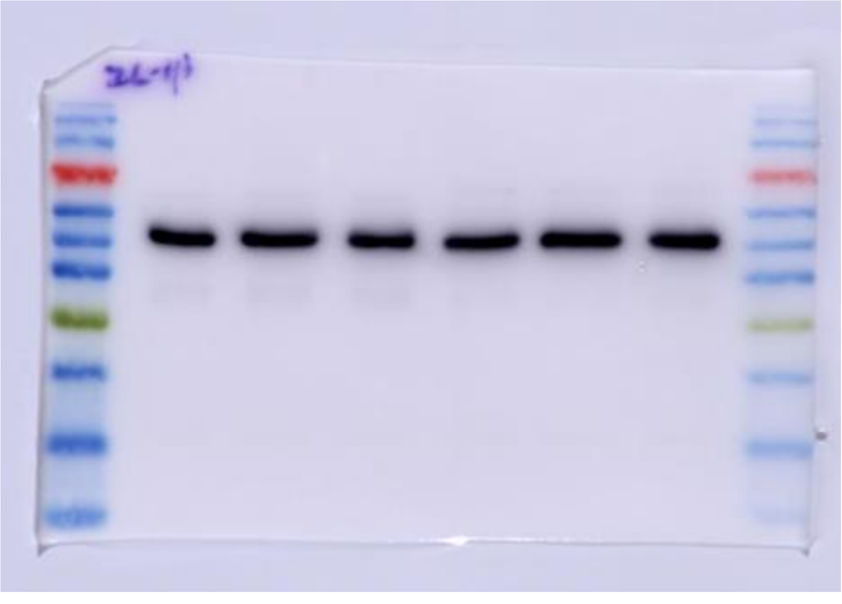

β-actin
