# Supplementary figures and images for: BNIP3-mediated mitophagy aggravates placental injury in preeclampsia via NLRP1 inflammasome
Source: Front Immunol. 2025 Apr 2;16:1530015. doi: 10.3389/fimmu.2025.1530015 (PMC11999839; doi:10.3389/fimmu.2025.1530015)

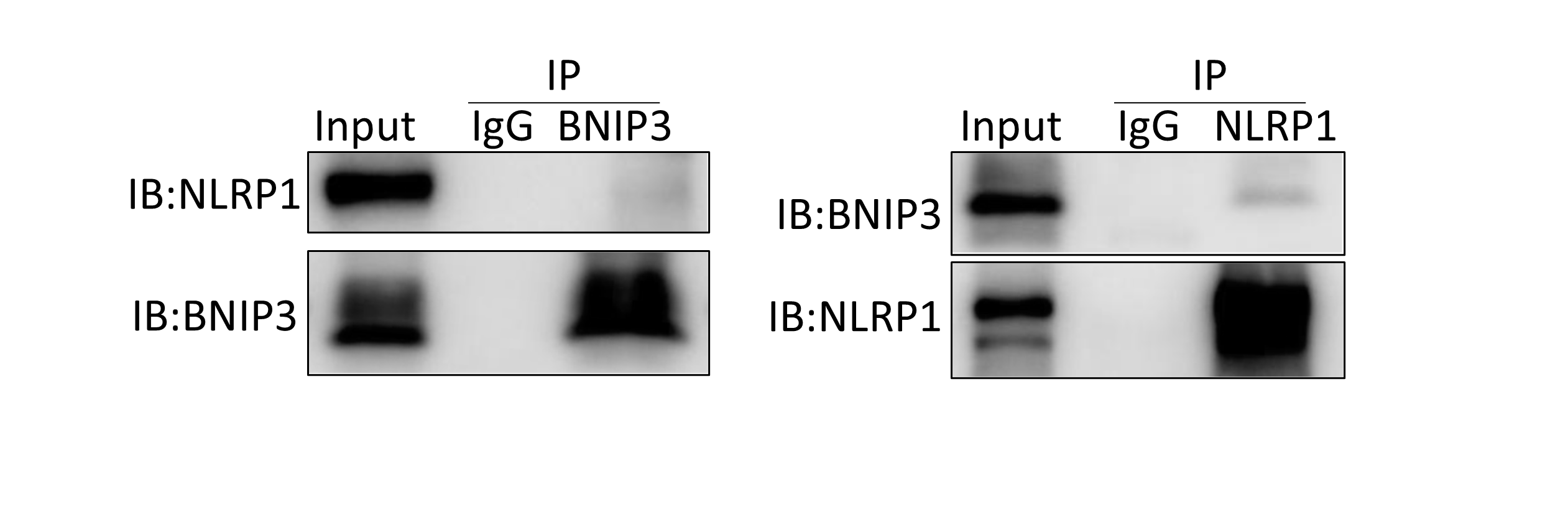

Supplement: Supplementary file 3 [file Image1.tif]
