# Supplementary material for: BNIP3-mediated mitophagy aggravates placental injury in preeclampsia via NLRP1 inflammasome
Source: Front Immunol. 2025 Apr 2;16:1530015. doi: 10.3389/fimmu.2025.1530015 (PMC11999839; doi:10.3389/fimmu.2025.1530015)
Supplement: Supplementary file 4 [file Table1.docx]

|  | Normal pregnancy  (n = 15) | Early-onset preeclampsia  (n = 15) | *p*-value |
| --- | --- | --- | --- |
| Maternal age (y) | 28.6±3.7 | 31.8±4.9 | 0.0549 |
| Body mass index (kg/m^2^) | 23.5±4.7 | 24.0±3.6 | 0.7293 |
| Systolic blood pressure (mm Hg) | 111.1±7.5 | 157.4±11.5 | < 0.0001 |
| Diastolic blood pressure (mm Hg) | 66.9±8.2 | 99.9±12.7 | < 0.0001 |
| Urine protein (g/24 h) | ND | 3.6±1.6 | < 0.0001 |
| Gestational age at delivery (d) | 275.5±6.8 | 241.4±19 | < 0.0001 |

Table S1. Clinical characteristics of the pregnant women enrolled in this study

Data are presented as mean ± SD, and significant difference between groups was analyzed by Student’s t test.
